# Origin of the ^31^P NMR Chemical Shift in Lewis Acid Adducts of Triethylphosphine Oxide. Does the Gutmann–Beckett Method Relate to Lewis Acid Strength?

**DOI:** 10.1021/jacs.5c17621

**Published:** 2026-04-01

**Authors:** Alexander A. Kolganov, Maximillian Kling, Matthew P. Conley, Evgeny A. Pidko

**Affiliations:** † Inorganic Systems Engineering Group, Department of Chemical Engineering, Faculty of Applied Sciences, 2860 Delft University of Technology, Van der Maasweg 9, Delft 2629 HZ, The Netherlands; ‡ Department of Chemistry, 8790 University of California, Riverside, California 92507, United States

## Abstract

The Gutmann–Beckett method involves the reaction of a phosphine oxide with a Lewis acid, followed by measurement of the change in ^31^P NMR chemical shift (Δδ) relative to the free phosphine oxide. This is the most commonly used experimental method to assess Lewis acid strength in solution and on solid materials containing Lewis acid sites. This study describes the origin of the ^31^P NMR Δδ deshielding that occurs in triethylphosphine oxide (TEPO) adducts of Lewis acids. 57 Lewis acid adducts were studied using DFT methods. These models span typical three-, four-, and five-coordinate Lewis acids as well as models that approximate the coordination sphere of Lewis acid sites proposed to be present in heterogeneous materials. When a TEPO···Lewis acid adduct forms, electron density from the oxygen is transferred to the Lewis acid, which reduces the negative hyperconjugation from the oxygen to the σ*_P–C_ that weakens the P=O bond. Experimental and DFT studies show that the ^31^P NMR chemical shift deshields in TEPO···Lewis adducts because the most shielded δ_33_ component of the chemical shift tensor shifts dramatically downfield. This deshielding is correlated with the weakening of the P=O bond. Natural chemical shift (NCS) analysis shows that δ_33_ deshielding in Lewis acid adducts is due to coupling of the filled σ_P–C_ with the empty π*_P=O_, the LUMO of the TEPO fragment. This study connects the ^31^P NMR chemical shift, in particular the experimentally observable Δδ_33_, to P=O bond weakening. Thus, the Gutmann–Beckett method does not provide information on adduct formation energy, the more typically sought measure of Lewis acidity, but rather provides a different thermodynamic descriptor of Lewis acid strength in the weakening of the P=O bond.

## Graphical abstract

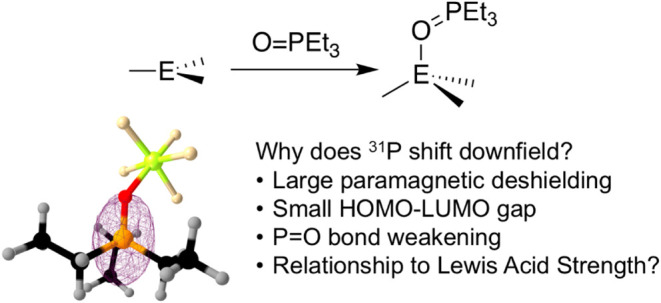

## Introduction

Lewis acids contain low lying empty orbitals that can accept electron density from substrates.^1^ This critically important interaction polarizes the bound substrate and facilitates further chemical reactivity. This model is used widely throughout organic synthesis to mediate bond forming reactions and to build molecular complexity.^2^ At the extreme, when a Lewis acid and Lewis base are too bulky to form the classical adduct, frustrated Lewis pair (FLP) behavior emerges, enabling activation of inert bonds, such as activation of the H–H bond in dihydrogen.^3^ Importantly, such reactivity can be achieved with main-group (often nonmetal) components, positioning FLPs as catalysts for transformations typically mediated by transition metals.^4^

Similar scenarios arise in materials that contain strong Lewis sites that are broadly important in heterogeneous catalysis.^5^ For example, aluminum oxide (Al_2_O_3_) is proposed to contain a low quantity of strong Lewis acid Al-sites that activate N_2_ or C–H bonds.^6^ These reactive sites may also play a role in generation of organometallic ion-pairs that form on Al_2_O_3_ supports.^7^ AlCl_x_F_3–x_ is proposed to contain stronger Lewis sites than Al_2_O_3_, which react with R_3_SiH to form electrophilic silylium-like intermediates that activate C–F bonds.^8^ Discrete silylium-like ions supported on weakly coordinating oxides show similar reactivity.^9^

Strong Lewis acids are also involved in reactions catalyzed by metal-exchanged zeolites^10^ or amorphous oxides containing well-defined metal sites.^11^ These materials can coordinate and heterolytically split aliphatic C–H bonds,^12^ resulting in organometallic intermediates that are relevant to propane dehydrogenation,^12c,13^ and ethylene polymerization.^14^ Though limited, this set of examples points to Lewis acidity as the unifying motif across reactivity encountered in solution and on surfaces.

The ability to control the reactivity of a Lewis acid is related to its strength. IUPAC defines Lewis acid strength as a measure of, “the equilibrium constants for Lewis adduct formation of a series of Lewis acids with a common reference Lewis base.”^15^ In practice, a common experimental method to assess Lewis acid strength was proposed by Gutmann and Beckett, which involves the reaction of a Lewis acid with triethylphosphine oxide (TEPO) (eq 1).^16,17^ TEPO often binds irreversibly to strong Lewis acids, and the resulting change in the isotropic ^31^P NMR chemical shift (Δδ_iso_) of the diamagnetic adduct relative to free TEPO is widely taken as a measure of Lewis acid strength. Care must be taken to ensure that the ^31^P NMR chemical shift measured in solution is representative of the static TEPO adduct and not affected by exchange between free and complexed TEPO.^17,18^


1

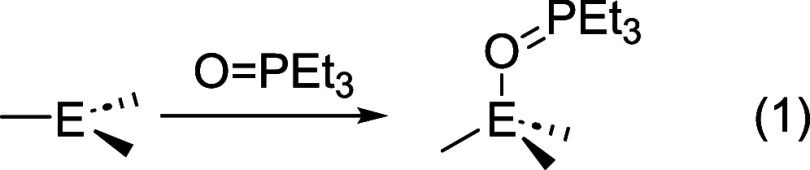



This method is broadly applied in solution and to solids containing strong Lewis or Brønsted acid sites.^19^ Lewis sites present on heterogeneous surfaces are obviously impossible to isolate and study crystallographically, thus indirect probes are essential to assess their Lewis acid strength. The Gutmann–Beckett method is very convenient for this purpose because solid-state ^31^P­{^1^H} MAS NMR spectra are generally easy to acquire. Additionally, TEPO adsorbed on surfaces does not generally engage in equilibria and/or solvent dependent dynamic behavior that can complicate studies in solution.^20^

However, Δδ_iso_ values across chemically diverse Lewis acids correlate poorly with calculated energies for adduct formation,^17^ suggesting that this method may not directly probe Lewis acid strength. In contrast, Brønsted acid strength correlates quite well with Δδ_iso_ of TEPO.^21^ In the absence of water, protons form linear hydrogen bond adducts with anions and/or hydrogen bond acceptors.^22^ TEPO is an H-bond acceptor and forms TEPO···H···X^–^, where X^–^ is the conjugate base of the HX acid. The key difference between TEPO···H···X^–^ and TEPO···LA is that a Lewis acid must undergo structural distortion to form the adduct that is a significant component of binding energy (Δ*E_bind_*) in this reaction. Erdmann and Greb proposed that Δ*E_bind_* should be viewed as the sum of the unfavorable deformation energy (*E_D_*) to distort the Lewis acid and the favorable interaction energy (*E_I_*) to form the adduct (eq 2).^17^ This treatment gives a good correlation between calculated *E_I_* and Δδ_iso_ (*R* ^2^ = 0.81).^17^

2

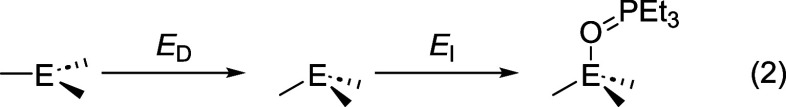




The NMR chemical shift relates to electronic structure,^23^ thus Δδ_iso_ values are reporting changes in electronic structure at phosphorus in TEPO···Lewis adducts. We set out to determine the origin of Δδ_iso_ in a broad set of Lewis acids shown in Figure 1. These span typical three-, four-, and five-coordinate Lewis acids to more modern Al­(OR^F^)_3_ (OR^F^ = C­(CF_3_)_3_),^24^ B­(*o*Cb)_3_ (*o*Cb = *ortho*-carborane),^25^ or HB^Me^ *o*Cb_2_.^26^ This study also includes cationic Lewis acids, including carbocations, silylium, germylium, stannylium, B­(Mes)_2_^+^,^27^ and Sb­(C_6_F_5_)_4_^+^;^28^ as well as transition metal containing Lewis acids. Eight surface models extend the analysis to representative Lewis acidic motifs in heterogeneous catalysts. These include models that approximate the coordination environment of strong aluminum^29^ or boron^30^ Lewis acids supported on amorphous silica, the pyrosulfate supported on zirconium oxide,^31^ and four cluster models of metal-exchanged zeolite models in the BEA topology.^32^ This breadth allows us to test the generality of the Gutmann–Beckett metric across chemically diverse bonding regimes and environments. Our analysis reveals that Δδ_iso_ primarily reflects adduct-induced polarization of the TEPO P=O bond, rather than the intrinsic strength of the TEPO···Lewis acid interaction.

**Figure 1:**
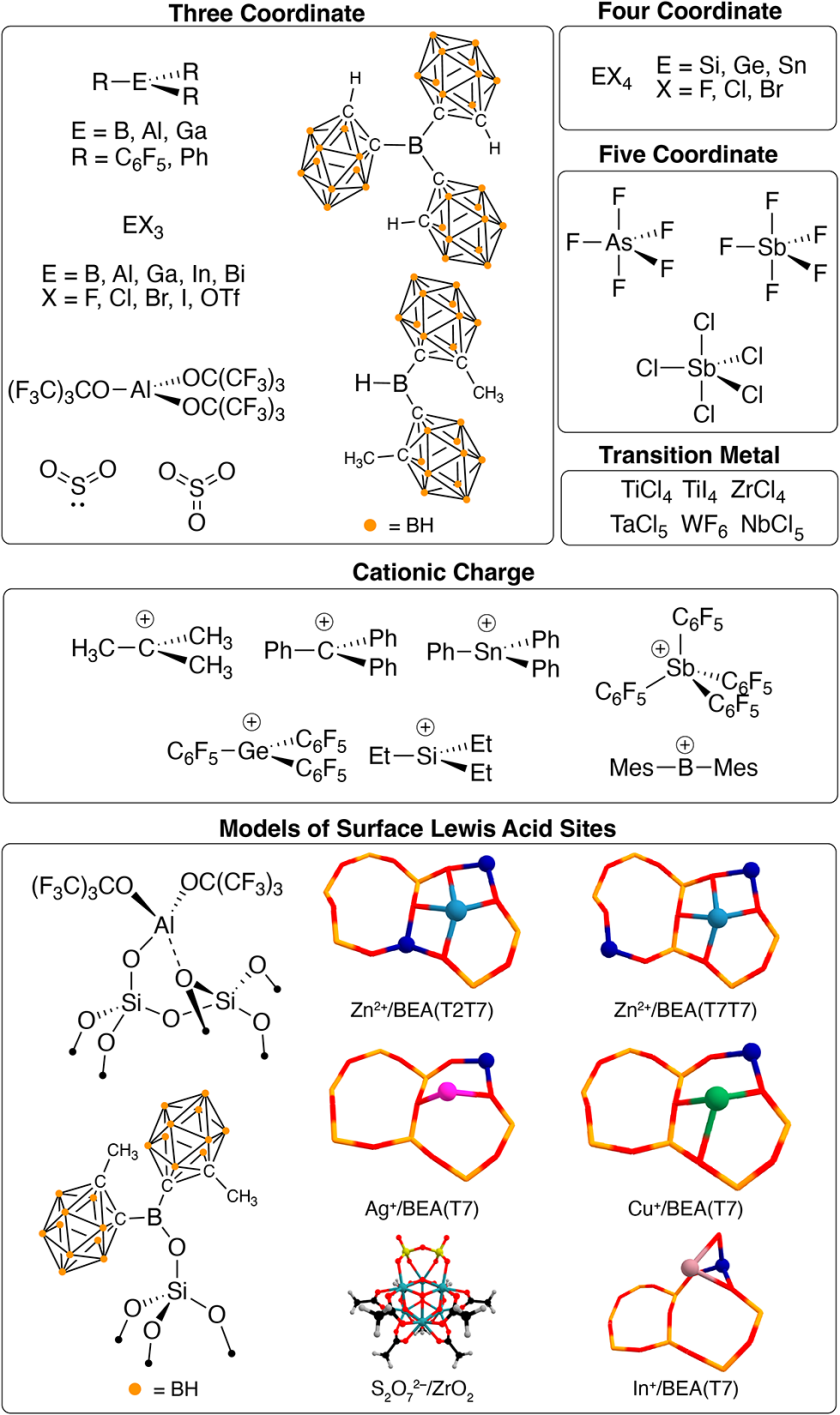
Lewis acids studied here.

## Results and Discussion

### Energetics of TEPO Adduct Formation

Figure 2 shows the natural localized molecular orbitals (NLMOs)^33^ that describe the bonding between phosphorus and oxygen in TEPO in NBO 7.0 formalism.^34^ The σ_P–O_ (not shown) is a dative σ bond originating from donation of the nonbonding lone pair on phosphorus to oxygen that completes the octet and results in three lone pairs on O. The n_1_(O) lone pair is colinear with the P–O bond and is localized on oxygen with high s-character (64% s, 36% p). Two lone pairs, n_2_(O) and n_3_(O), orient roughly perpendicular to the P–O bond axis and donate electron density to their respective σ*_P–C_ antibonding orbitals (Figure 2a). These NLMOs display occupancy on both the oxygen and the corresponding antibonding P–C regions. This analysis is in agreement with previous computations,^35^ and is referred to as negative hyperconjugation.^36^

**Figure 2:**
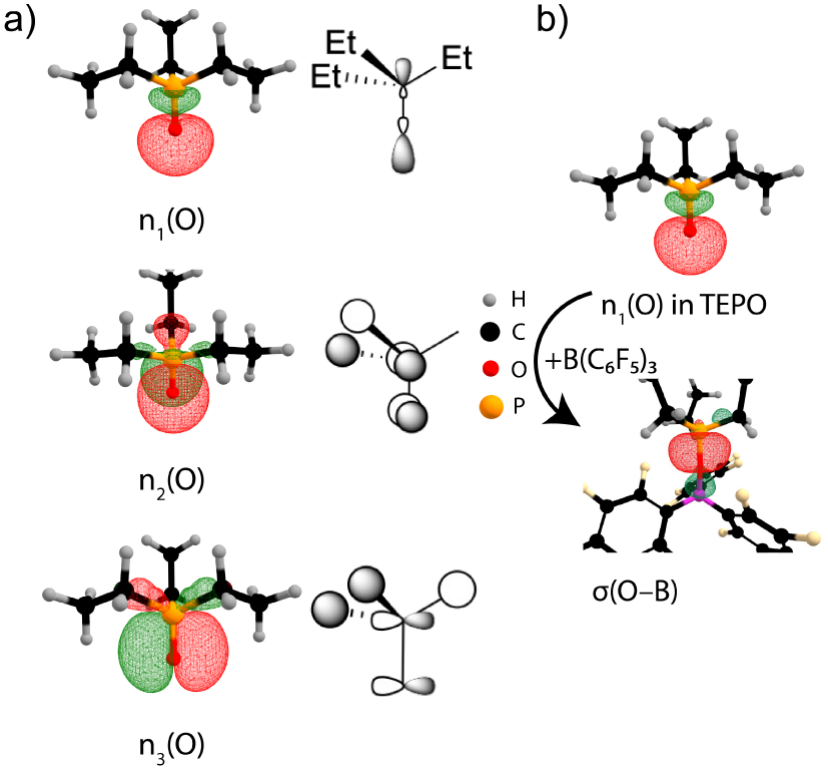
(a) Natural-localized molecular orbital (NLMO) plots of negative hyperconjugation in free TEPO. ChemDraw sketches of these orbitals are given next to the NLMO plots. (b) Coordination to B­(C_6_F_5_)_3_ converts the n_1_(O) lone pair into an O → B σ-bond.

To streamline the discussion, we will largely focus on free TEPO and the representative TEPO···B­(C_6_F_5_)_3_ adduct to illustrate how a Lewis acid perturbs the electronic structure at phosphorus, and to show how this relates to the ^31^P NMR chemical shift. Other Lewis acids shown in Figure 1 follow the trends described below for TEPO and the TEPO···B­(C_6_F_5_)_3_ adduct. All relevant data for those Lewis acids is summarized in the Section S7 of the Supporting Information.

The n_1_(O) lone pair is responsible for adduct formation with Lewis acids. At the ωB97M-D3BJ/def2-TZVPP level of theory the calculated binding energy (Δ*E_bind_*) in TEPO···B­(C_6_F_5_)_3_ adduct is −28.2 kcal mol^–1^ (Table 1). From eq 2, Δ*E_bind_* should be viewed as the sum of the distortion energy (*E_D_*) and the interaction energy (*E_I_*). The optimized structure of TEPO···B­(C_6_F_5_)_3_ has a B–O–P bond angle of 156.1°, close to the experimentally observed 161.0°.^37^ The NLMO describing this interaction is shown in Figure 2b.

**Table 1:** Calculated Binding (Δ ***E*** *_**bind**_*) and Deletion (Δ ***E*** ^(***del***)^) Energies for TEPO TEPO···B­(C_6_F_5_)_3_ ^a^

	Δ*E_bind_*	$$\Delta E_{n_{x}\left(O\right)\to 3\times \sigma *\left(P-C\right)}^{\left(del\right)}$$				$\Delta E_{O\to \{P,C\}}^{\left(del\right)}$ ^d^
Compound		1^b^	2^b^	3^b^	all^c^	
TEPO	-	2.0	35.9	37.7	86.3	182.2
TEPO·B(C_6_F_5_)_3_	–28.2	-	19.9	20.7	44.1	93.4

a All Energies are in kcal mol^–1^.

b Deletion energies for removal of individual n­(O) orbitals.

c Combined deletion energy when all n­(O) orbitals are removed.

d Deletion energy for all donor–acceptor interactions from O to the P and C atoms bonded to P.

The σ donation from O to B reduces lone pair/lone pair repulsion and weakens the negative hyperconjugation from n_2_(O) and n_3_(O). Quantitatively, this effect can be assessed with deletion energies (Δ*E* ^(*del*)^) using eq 3:

3 $$\Delta E_{i,j}^{\left(del\right)}=E\left[F^{\left(i,j\right)}=0\right]-E\left[F^{\left(full\right)}\right]$$

Δ*E* ^(*del*)^ is the total energy increase when selected Fock matrix elements (*F* ^(*i*,*j*)^) corresponding to selected donor–acceptor couplings are removed followed by a full SCF recalculation of the electronic structure at fixed geometry.^38^

We calculated two types of energy deletions:

a)
$\Delta E_{n_{x}\left(O\right)\to 3\times \sigma *\left(P-C\right)}^{\left(del\right)}$ is the individual lone pair deletion energy from removal of the donor–acceptor interactions between an oxygen lone pair (n_1_(O), n_2_(O), or n_3_(O)) and the three σ*­(P–C) antibonding orbitals. This provides a selective measure of the n­(O) → σ*­(P–C) hyperconjugative stabilization. Combined lone pair deletion energy from the simultaneous removal of all oxygen lone pair donor–acceptor interactions to σ*­(P–C) orbitals was also calculated because individual deletion energies are nonadditive.
b)
$\Delta E_{O\to \{P,C\}}^{\left(del\right)}$ is the deletion energy of all Fock matrix elements between the oxygen atom and the phosphorus/carbon atoms bonded to P. This removes all occupied → vacant orbital interactions originating from oxygen and terminating at P or the three carbon atoms, which removes the total influence of oxygen lone pair delocalization on molecular stability and includes n­(O) → σ*­(P–C) delocalization.

In the case for free TEPO, the combined lone pair deletion energy 86.3 kcal mol^–1^, with the nearly identical contributions from n_2_(O) and n_3_(O) of 35.9 and 37.7 (Table 1). The formation of TEPO···B­(C_6_F_5_)_3_ adduct results in a significant reduction of the n­(O) → σ*­(P–C) delocalization, as is evidenced from the decrease of the combined deletion energy to 44.1 kcal mol^–1^ as well as the individual contributions from n_2_(O) and n_3_(O) (19.9 and 20.7 kcal mol^–1^, respectively). This result indicates, somewhat unsurprisingly, that Lewis acid coordination to TEPO results in P=O bond weakening, which is a thermochemical descriptor of Lewis acid strength.

As expected, $\Delta E_{O\to \{P,C\}}^{\left(del\right)}$ for TEPO is larger than the combined lone pair delocalization because it captures additional minor channels beyond n­(O) → σ*­(P–C). Nevertheless, this interaction remains dominant, as evidenced by the orbital occupancy changes upon deletion. The natural populations of n_2_(O) and n_3_(O) each increase by 0.18 electrons, while the three σ*­(P–C) orbitals each decrease by 0.09 electrons; all other orbital population shifts are ≤ 0.01 electrons. This confirms that n­(O) → σ*­(P–C) hyperconjugation accounts for the majority of the stabilization captured by deletion. For the analysis presented below we use $\Delta E_{O\to \{P,C\}}^{\left(del\right)}$ because this is the most inclusive descriptor of negative hyperconjugative interactions in the phosphine oxide adducts. (Figure S5.4) $\Delta E_{O\to \{P,C\}}^{\left(del\right)}$ , and related individual and combined deletion energies for all adducts are given in the Supporting Information (Table S7.1).

In all adducts Δ*E_bind_* are all exoergic and $\Delta E_{O\to \{P,C\}}^{\left(del\right)}$ values decrease relative to free TEPO. There is no obvious correlation between Δ*E_bind_* and $\Delta E_{O\to \{P,C\}}^{\left(del\right)}$ (Figure S4.1b) The strongest Lewis acid in this series in terms of Δ*E_bind_* is the Zn^2+^/BEA (T7T7) model (Δ*E_bind_* = −81.2 kcal mol^–1^). The large Δ*E_bind_* does not imply a more pronounced weakening of the coordinated P=O bond. The $\Delta E_{O\to \{P,C\}}^{\left(del\right)}$ energy in TEPO···Zn^2+^/BEA (T7T7) is 93.6 kcal mol^–1^, close to $\Delta E_{O\to \{P,C\}}^{\left(del\right)}$ of B­(C_6_F_5_)_3_.

The primary coordination sphere of the Zn^2+^ ion in the Zn^2+^/BEA (T7T7) is shown in Figure 3a. Zinc is in a distorted square planar coordination environment, displaced from the plane defined by the four oxygens by 0.50 Å. One Zn–O bond is significantly longer than the other three Zn–O bonds, suggesting this bond is weaker than the other three. The Lewis acidity of the Zn^2+^ center is further enhanced because one of the two charge-compensating AlO_4_^–^ tetrahedra resides in an adjacent ring rather than in the ring directly coordinating Zn^2+^. As a result, the positive charge on zinc is less effectively compensated, and the metal coordinates predominantly to less basic Si–O–Si oxygens rather than Si–O–Al ones. A molecular analogy could be zinc salen complexes, which also adopt distorted square planar coordination environments and are strong Lewis acids that readily bind a fifth ligand in supramolecular assemblies.^39^

**Figure 3:**
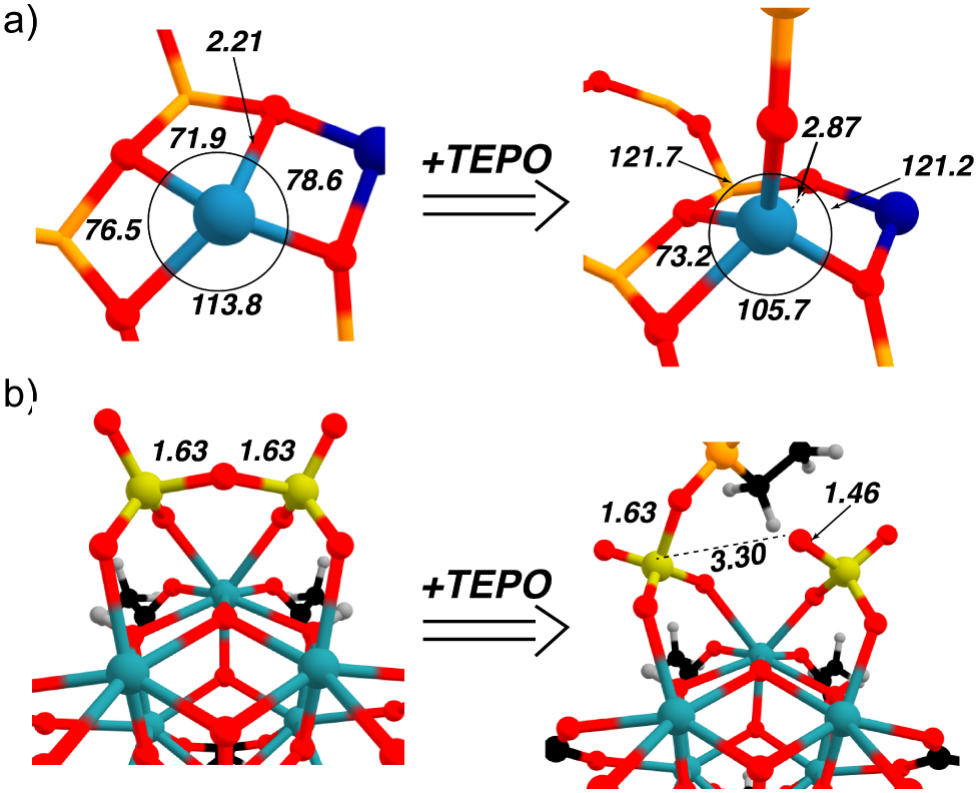
Local structures of Zn^2+^/BEA (T7T7) (a) and S_2_O_7_^2–^/ZrO_2_ cluster (b) and their TEPO adducts. Important bond angles and distances (in Å) are listed in the figure.

Coordination of TEPO results in three similar Zn–O bond distances to the zeolitic framework and O–Zn–O bond angles near tetrahedral values. The weak Zn–O bond in Zn^2+^/BEA (T7T7) is no longer present, the distance between zinc and this oxygen increases to 2.87 Å. These results explain why Δ*E*_bind_ is large and $\Delta E_{O\to \{P,C\}}^{\left(del\right)}$ is small. The distorted square planar Zn^2+^ center is a strong Lewis acid that relaxes to the more common, and less Lewis acidic, tetrahedral zinc when TEPO coordinates to the metal. Another aspect that likely contributes to the large Δ*E*_*bind*_ is relaxation of the strained 5-membered ring. TEPO coordination also results in O–Al–O bond angle relaxing from 91.1° in Zn^2+^/BEA (T7T7) to 100.0°, the latter of which is closer to tetrahedral values associated with SiO_x_ and AlO_x_ nodal points in zeolitic materials.

A small Δ*E*_bind_ does not imply the P=O bond is strong (e.g., large $\Delta E_{O\to \{P,C\}}^{\left(del\right)}$ energies). The S_2_O_7_^2–^/ZrO_2_ cluster (Figure 3b) has Δ*E_bind_* of only −12.8 kcal mol^–1^, but very small $\Delta E_{O\to \{P,C\}}^{\left(del\right)}$ energy of 58.3 kcal mol^–1^, the lowest in the series studied here. This adduct forms by opening the pyrosulfate to form a sulfate anion and SO_3_ bound to Zr that coordinates to TEPO.^31^ In this case the Δ*E*_bind_ is a composite of S–O bond breaking (endothermic) and S–O=P bond making (exothermic), which likely contributes to the low binding energy using this model.

The calculated TEPO adducts adopt a range of structures that contain E–O–P bond angles ranging from 179.8° to 110.8°. There is no obvious trend in the data connecting the E–O–P bond angles to $\Delta E_{O\to \{P,C\}}^{\left(del\right)}$ or the ^31^P NMR Δδ_iso_ discussed later. Because of the large range of E–O–P bond angles, about half of the adducts contain three lone pairs from NLMO analysis; the third lone pair is assigned to n_1_(O) from TEPO that is not completely transferred to the Lewis acid.

Adducts with linear E–O–P bond angles (>175°, AlPh_3_, B­(oCb)_3_, Sb­(C_6_F_5_)_4_^+^, Ge­(C_6_F_5_)_3_^+^, and TiI_4_) are essentially C_3v_ symmetric. The n_1_(O) lone pair donates to an empty orbital on the Lewis acid and the individual lone pair energies for n_2_(O) and n_3_(O) are nearly degenerate. However, there are weak interactions between n_2_(O) and n_3_(O) and the Lewis acid. The NLMOs for n_2_(O) and n_3_(O) in TEPO···Ge­(C_6_F_5_)_3_^+^ are shown in Figure 4a, showing that both donate electron density to low-lying σ* orbitals on germanium. NBO analysis shows that n_3_(O) has 11% Ge characters.

**Figure 4:**
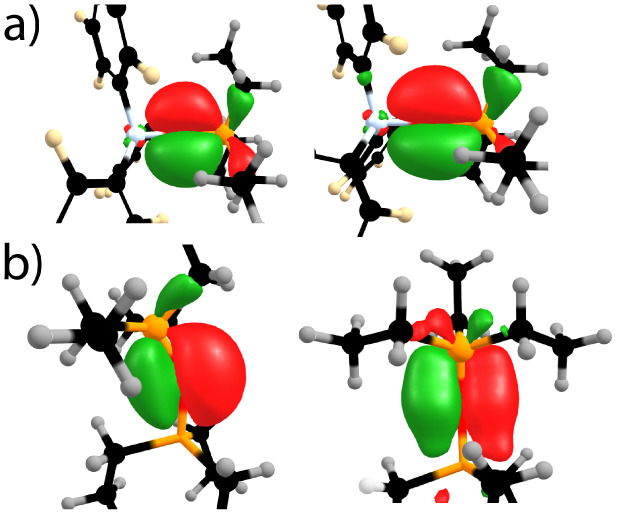
NLMO plots of n_2_(O) (left) and n_3_(O) (right) for Ge­(C_6_F_5_)_3_^+^ (a) and Et_3_Si^+^ (b).

As the E–O–P bond angle contracts one large (n_2_(O)) and one small (n_3_(O)) lone pair deletion energy is obtained. In most cases, n_3_(O) also interacts with the Lewis acid. Figure 4b shows the n_2_(O) and n_3_(O) NLMOs for TEPO···Et_3_Si^+^. The n_3_(O) interacts with σ* orbitals, but n_2_(O) orients away from the σ* orbitals in Et_3_Si^+^. NBO analysis shows that n_3_(O) contains 12% silicon character. This trend extends to the other Lewis acids in our extended data set (Figure 1), and explains why there is one large and one small lone pair deletion energy for the π-orbital lone pairs in TEPO adducts. There are a few exceptions, however. First, due to geometric restriction in Ag^+^/BEA, Cu^+^/BEA, and In^+^/BEA, TEPO orients n_3_(O) to the Lewis site in the adduct, leaving n_1_(O) unperturbed (Figure S6.1). Second, transition metals accept electron density from these orbitals with empty d_π_ orbitals, as expected.

### Origin of the ^31^P NMR Chemical Shift in Free TEPO and TEPO Adducts

For an NMR active nucleus, the chemical shift is derived from a second rank tensor (δ_11_, δ_22_, δ_33_) that averages in solution due to rapid tumbling to give the isotropic chemical shift (δ_iso_ = (δ_11_ + δ_22_ + δ_33_)/3). These terms are directly related to shielding (σ_ii_) experienced by the nucleus, referenced to a standard (σ^iso^) using eq 4:

4 $$\left|\begin{array}{lll} \delta_{11} & 0 & 0\\ 0 & \delta_{22} & 0\\ 0 & 0 & \delta_{33} \end{array}\right|=\sigma_{iso}^{ref}\left|\begin{array}{lll} 1 & 0 & 0\\ 0 & 1 & 0\\ 0 & 0 & 1 \end{array}\right|-\left|\begin{array}{lll} \sigma_{11} & 0 & 0\\ 0 & \sigma_{22} & 0\\ 0 & 0 & \sigma_{33} \end{array}\right|$$

The ^31^P­{^1^H} magic angle spinning (MAS) NMR spectrum of TEPO and TEPO···B­(C_6_F_5_)_3_ is shown in Figure 5 and Table 2 summarizes experimental and calculated ^31^P NMR tensor data for these compounds. The isotropic chemical shift of free TEPO is 49.4 ppm, which is flanked by spinning sidebands that appear at integer multiples of the rotor spinning frequency (ν_rot_ = 3 kHz). The intensities of the spinning sidebands relate to the δ_ii_ values from the chemical shift tensor in eq 4. Simulation of the experimental spectrum is also shown in Figure 5a and gives (δ_11_, δ_22_, δ_33_) = (112.5, 102.6, −67.0). Similar tensor values were reported previously.^40^ At the PBE0/pcsSeg-2//ωB97M-D3BJ/def2-TZVPP level of theory TEPO has predicted NMR parameters with an isotropic chemical shift of 42.3 ppm and (δ_11_, δ_22_, δ_33_) = (112.4, 111.6, −97.3). These values are referenced to ^31^P NMR chemical shifts for a variety of phosphines and phosphine oxides calculated at the same level of theory (Figure S3.1). The discrepancy in δ_33_ values between theory and experiment is related to the differences in monomeric TEPO, used in the calculation, and the chemical environment encountered in the crystal used for the experimental solid-state NMR measurement. Indeed, the X-ray crystal structure of TEPO shows that each P=O has close contacts to three C–H bonds from a neighboring TEPO.^41^ Modeling the chemical shift of a periodic model using the coordinates from the experimental structure at the PBE/650 eV//PBE0-rVV10/DZVP-MOLOPT-SR-GTH level of theory, using a crystal structure optimized with nonlocal dispersion corrections, predicts an isotropic chemical shift of 55.5 ppm and (δ_11_, δ_22_, δ_33_) = (112.8, 113.9, −65.3) that more accurately reproduces the δ_33_ value. These values were referenced to ^31^P NMR chemical shifts of PPh_3_ molecular crystal (Table S1.1).

**Figure 5:**
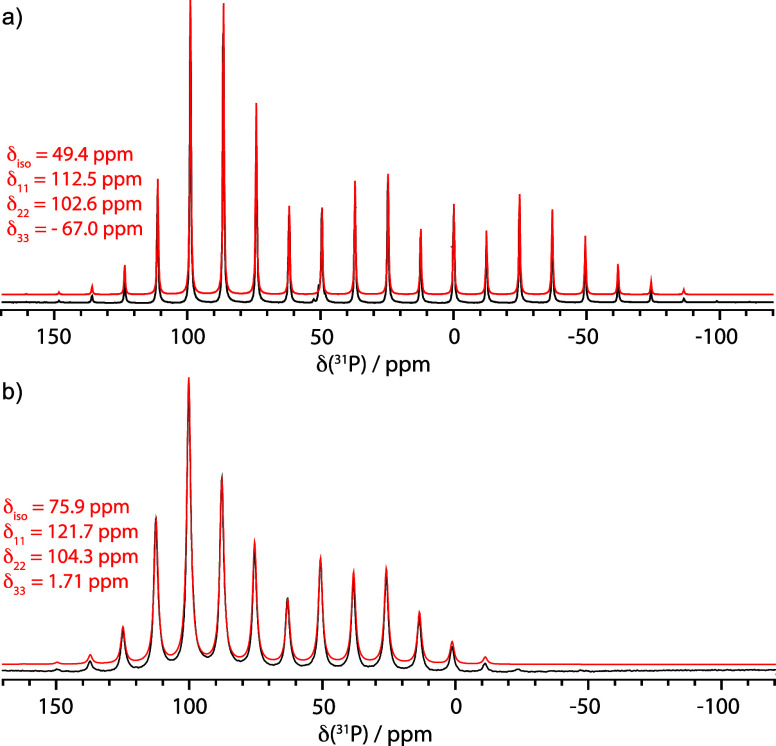
^31^P­{^1^H} MAS NMR spectra TEPO (a) and TEPO···B­(C_6_F_5_)_3_ (b). The experimental data is shown in black, and the simulated spectrum is shown in red.

**Table 2:** Experimental and Calculated NMR Parameters for TEPO and TEPO···B­(C_6_F_5_)_3_ Reported in ppm

	Compound	δ_iso_	δ_11_	δ_22_	δ_33_
1	TEPO (exp)	49.4	112.5	102.6	–67.0
2	TEPO (QM-Mono)^a^	42.3	112.4	111.6	–97.3
3	TEPO (QM-p)^b^	55.5	112.8	113.9	–65.3
4	TEPO···B(C_6_F_5_)_3_ (exp)	75.9	121.7	104.3	1.7
5	TEPO···B(C_6_F_5_)_3_ (QM-Mono)^a^	71.3	116.4	103.9	–6.4

a NMR values from the monomeric model calculated at the PBE0/pcsSeg-2//ωB97M-D3BJ/def2-TZVPP level of theory.

b Calculated as a periodic model using the close contacts present in the X-ray crystal structure of TEPO at the PBE/650 eV//PBE0-rVV10/DZVP-MOLOPT-SR-GTH level of theory.

Figure 5b shows solid-state ^31^P­{^1^H} NMR data for TEPO···B­(C_6_F_5_)_3_. The isotropic chemical shift is 75.9 ppm, corresponding to a Δδ of 26.5 ppm, close to the Δδ obtained in CD_2_Cl_2_ solution (26.6 ppm).^42^ The simulated spectrum gives (δ_11_, δ_22_, δ_33_) = (121.7, 104.3, 1.7). DFT calculations at the PBE0/pcsSeg-2//ωB97M-D3BJ/def2-TZVPP level of theory predicts Δδ of 29.0 ppm and (δ_11_, δ_22_, δ_33_) = (116.4, 103.9, −6.4), close to experimental values. These data show that δ_11_ and δ_33_ increase with respect to the calculated values for free monomeric TEPO, with δ_33_ shifting far more dramatically than δ_11_.

Calculated NMR tensor parameters for the complete selection of Lewis acids in Figure 1 are given in Table S7.1. These values were calculated as the difference in shielding constants between free TEPO and the TEPO···Lewis acid adduct These calculated Δδ_iso_ generally agree well with available experimental data. There is no correlation between Δδ_iso_ versus Δ*E_bind_* of adduct formation.^17,43^ In all adducts studied here, Δδ_33_ shifts to significantly more deshielded values than Δδ_11_ or Δδ_22_. Figure 6a contains simulated static ^31^P NMR tensors for selected Lewis acids to illustrate this trend.

**Figure 6:**
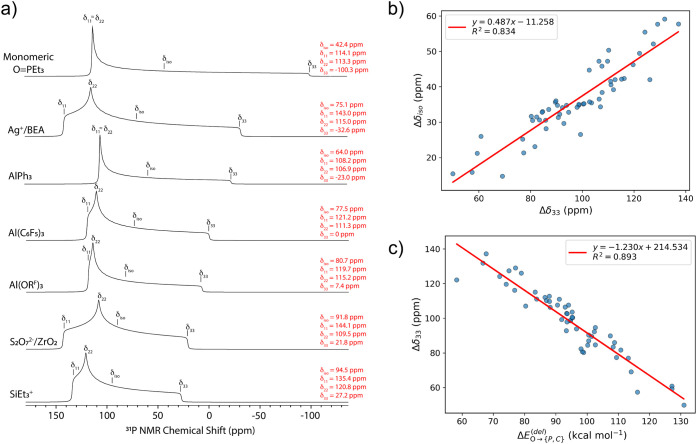
(a) Simulations of the calculated ^31^P NMR tensors for TEPO adducts of selected Lewis acids. The tensors were simulated using TopSpin 4.4.1 and shown with 250 Hz of line broadening to approximate the static line shape that would be obtained experimentally. The tensor positions for each component (δ_ii_; i = 1, 2, 3) and isotropic chemical shift (δ_iso_) are marked in each ^31^P NMR tensor. The numerical values for each of these components are listed in red. (b) Plot of the change in isotropic chemical shift (Δδ_iso_) and the change in δ_33_ (Δδ_33_). (c) Plot of Δδ_33_ versus $\Delta E_{O\to \{P,C\}}^{\left(del\right)}$ energies showing that weakening of n­(O) → σ*­(P–C) hyperconjugation is the primary electronic origin of δ_33_ deshielding.

A plot of Δδ_33_ versus Δδ_iso_ is linear (Figure 6b, *R* ^2^ = 0.82), showing that this term in the chemical shift tensor correlates with the isotropic chemical shift. Plots of Δδ_11_ or Δδ_22_ versus Δδ_iso_ correlate poorly (Figure S5.1a–b). This is somewhat unusual because the most deshielded δ_11_ component of the chemical shift tensor is often responsible for downfield NMR chemical shifts.^23b,44,45,46^ Plots of Δδ_11_ or Δδ_22_ versus $\Delta E_{O\to \{P,C\}}^{\left(del\right)}$ energies are not linear (Figure S5.3a,b). A plot of Δδ_33_ versus the $\Delta E_{O\to \{P,C\}}^{\left(del\right)}$ is linear (Figure 6c, *R* ^2^ = 0.893), indicating that these parameters are related.

The unreferenced chemical shielding tensor shown in eq 4 can be decomposed into a diamagnetic term (σ^d^), a result of the effect of **B**_**0**_ on the ground-state wavefunction of the nucleus, and a paramagnetic term (σ^p^) that results in deshielding (eq 5).^23a^

5 $$\sigma =\sigma^{d}+\sigma^{p}$$

Diamagnetic shielding does not correlate with chemical shift (Figure S5.2a). The Δδ_33_ values shown in Figure 6 are related to σ^p^ (Figure S5.2b).

The magnitude of σ^p^ is related to the coupling of a ground state wave function (ϕ_0_) to an excited state wave function (ϕ_n_) through the angular momentum operator (L̂*_ki_*, where *ki* = element of the shielding tensor, eq 6).^47^

$$\sigma_{ij}^{para}\propto \frac{\left\langle \varphi_{0}|{\mathrm{L̂}}_{ki}|\varphi_{n}\right\rangle \left\langle \varphi_{n}\left|\frac{{\mathrm{L̂}}_{kNj}}{r_{kN}^{3}}\right|\varphi_{0}\right\rangle }{\Delta E_{n-0}}$$ 6

The denominator in eq 6 indicates that if ϕ_0_ and ϕ_n_ are close in energy a large σ^p^ contribution is expected.^23b,48^ Thus, eq 6 connects NMR properties to the orientation of the magnetic shielding tensor, and in turn provides information about the electronic structure at phosphorus. The orientation of the magnetic shielding tensors for TEPO and TEPO···B­(C_6_F_5_)_3_ at the ZORA-PBE0/pcSseg-2//ωB97M-D3BJ/def2-TZVPP level of theory are shown in Figure 7. Both tensors are aligned such that σ_11_ and σ_22_ are perpendicular to the P–O bond, while σ_33_ is aligned with the P–O bond. This alignment is conserved across all Lewis adducts.

**Figure 7:**
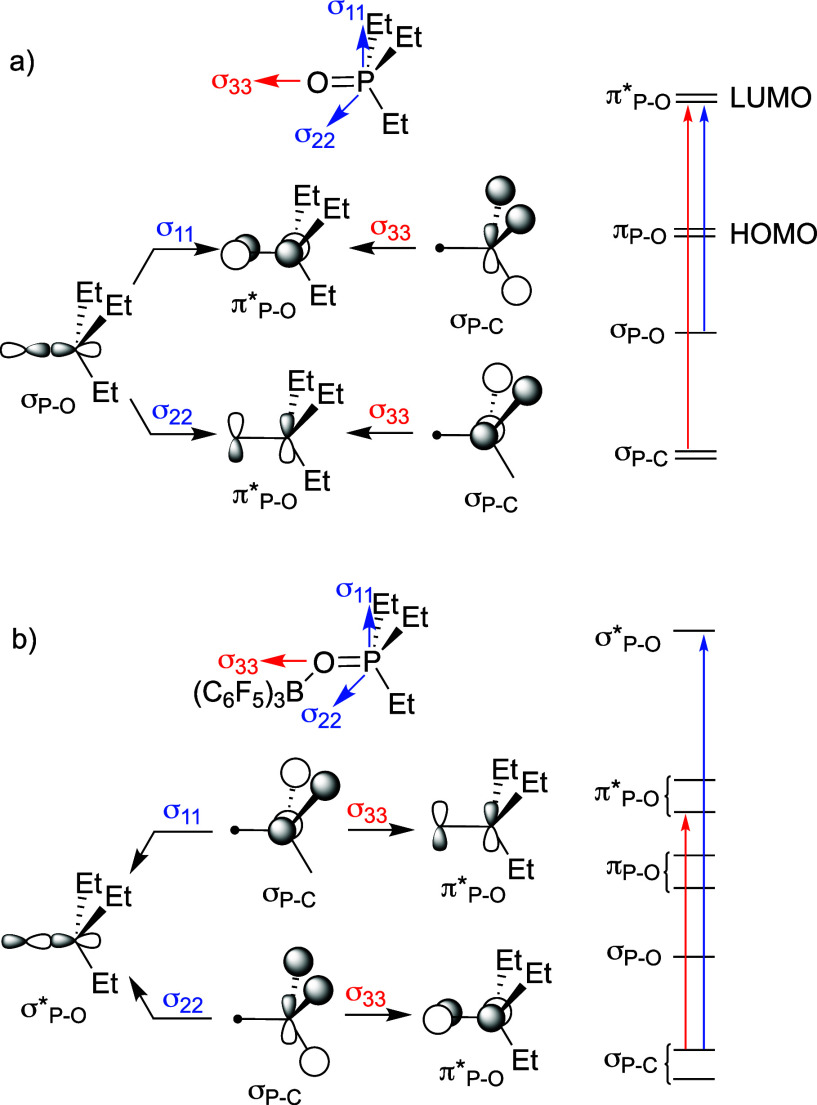
Orientation of the chemical shielding tensors and orbitals involved in σ^p^ deshielding for free TEPO (a) and TEPO···B­(C_6_F_5_)_3_ (b).

The origin of (de)­shielding was studied using natural chemical shift (NCS) analysis. Quantitative NCS values for NLMO contribution to each shielding term for free TEPO and the Lewis adducts are given in Tables S7.4–S7.6. The qualitative results for free TEPO are shown in Figure 7a. The largest NLMO contributor to paramagnetic deshielding in σ_11_ and σ_22_ is the σ­(P–O) orbital. The largest NLMO contributors to σ_33_ are σ­(P–C) orbitals. Regardless of the contributor orbital, application of the angular momentum operator results in the formally π* combination, the LUMO of the molecule. The reason that σ_11_ and σ_22_ are more deshielded than σ_33_ is because the energy gap between σ­(P–O) and the π* is smaller than the energy gap between σ­(P–C) and the π*.

Because the tensors in TEPO and the TEPO···B­(C_6_F_5_)_3_ are aligned, a similar orbital scheme would be expected. However, NCS analysis shows that σ­(P–C) orbitals are the major contributors to paramagnetic deshielding for σ_11_, σ_22_, and σ_33_. In σ_11_ and σ_22_ the angular momentum operator couples the σ­(P–C) to σ*­(P–O) orbitals (Figure 7b). This is expected to have a large energy gap and minimal effects on deshielding. The σ­(P–O) continues to contribute to σ_11_ and σ_22_, though less than the σ_P–C_ orbitals. These two effects apparently balance one another and is an explanation of why these two components do not experience significant (de)­shielding when TEPO forms an adduct with a Lewis acid.

The orbitals involved in σ_33_ are the same for free TEPO and TEPO···B­(C_6_F_5_)_3_. The Lewis acid weakens the P=O bond and reduces the energies of the π* orbitals. This reduces the energy between the σ­(P–C) and π* orbitals, increases σ^p^ for σ_33_, results in deshielding of the ^31^P NMR signal, and is the origin of the relationship between Δδ_33_ and $\Delta E_{O\to \{P,C\}}^{\left(del\right)}$ shown in Figure 6c. These effects are amplified from donation of a second pair of electrons from a different oxygen lone pair into a low-lying σ* (or empty d_π_) orbital. Interactions between Lewis bases and low-lying σ* orbitals, often referred to as σ-hole interactions, can result in very strong Lewis acids.^49^ This breaks π* degeneracy and reduces the π/π* energy gap further.

The Δδ_iso_ is the most commonly reported value when assessing Lewis acidity. The correlation between Δδ_iso_ and $\Delta E_{O\to \{P,C\}}^{\left(del\right)}$ energies presented in Figure 8 is rather poor (*R* ^2^ = 0.75) compared to the correlation with Δδ_33_ because Δδ_11_ and Δδ_22_ do not systematically increase (or decrease) as a function of P=O bond weakening, which is related to the orbital scheme described in Figure 7. Clearly using only Δδ_iso_ could result in an underestimation of Lewis acidity. This is seen in particular for S_2_O_7_^2–^/ZrO_2_, which has the lowest $\Delta E_{O\to \{P,C\}}^{\left(del\right)}$ value in the series, but has a low Δδ_iso_ of 49.5 ppm.

**Figure 8:**
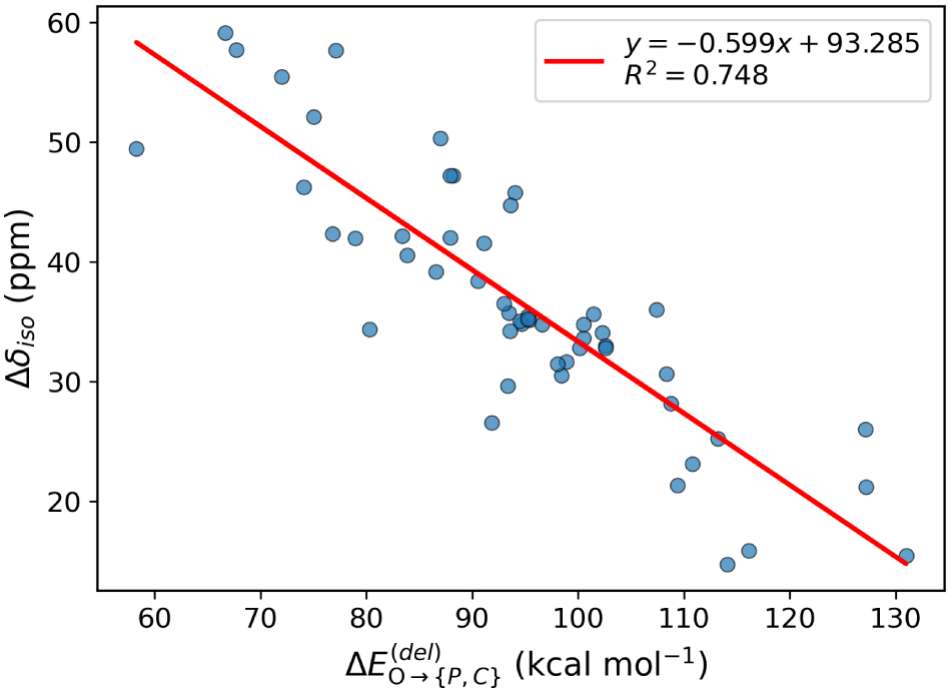
Plot of Δδ_iso_ versus $\Delta E_{O\to \{P,C\}}^{\left(del\right)}$ .

The results indicate that arguments about Lewis acidity when using the Gutmann–Beckett method should include solid-state NMR analysis. This is a considerable experimental demand that may not be readily available to many laboratories, and could be further complicated by isolation of potentially unstable species in the solid state. Rubini and coworkers showed that magnetic field dependent *T*_1_ relaxation rates in phosphine oxides and phosphine oxide adducts can deliver some information about the chemical shift tensor in solution.^50^ In particular, these measurements give the span (Ω) of the NMR chemical shift tensor, which is the difference between the most shielded and least shielded component of the NMR chemical shift tensor, eq 7:^51^

7 $$\Omega =\delta_{11}-\delta_{33}$$

In the adducts studied here, Ω generally decreases as Lewis acidity increases, but the correlation between Ω and $\Delta E_{O\to \{P,C\}}^{\left(del\right)}$ is weak (*R* ^2^ = 0.67, Figure S5.3c). This is also due to Δδ_11_ and Δδ_22_ that do not systematically increase (or decrease) as a function of P=O bond weakening. This issue can be corrected by approximating δ_33_ using eq 8:

8 $$\delta_{33}=\frac{3\delta_{iso}-\delta_{22}-\Omega }{2}$$

Though δ_22_ is unknown, the calculated Δδ_22_ values are generally small (Table S7.1). A plot of δ_33_, calculated using eq 8 but ignoring δ_22_ (i.e., δ_22_ = 0) is shown in Figure 9. Though individual δ_33_ are obviously inaccurate, the correlation implies that approximate $\Delta E_{O\to \{P,C\}}^{\left(del\right)}$ values can be obtained directly from solution ^31^P­{^1^H} NMR measurements, provided *T*_1_ measurements are acquired at multiple magnetic field strengths.

**Figure 9:**
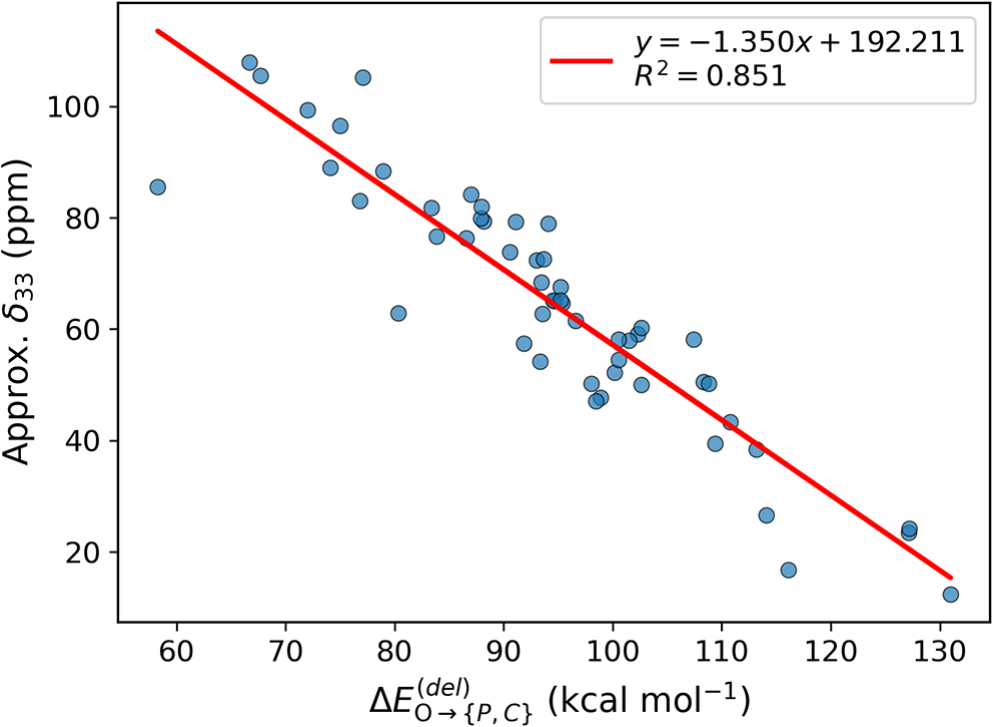
Plot of the approximate δ_33_ calculated with (8) versus $\Delta E_{O\to \{P,C\}}^{\left(del\right)}$ .

Figure 6c and Figure 9 connect experimental observables, obtained either from solid-state or solution ^31^P­{^1^H} NMR measurements, to $\Delta E_{O\to \{P,C\}}^{\left(del\right)}$ obtained from quantum chemical calculations. While $\Delta E_{O\to \{P,C\}}^{\left(del\right)}$ is an abstract value derived within the NBO formalism, its “real-world” meaning is straightforward: $\Delta E_{O\to \{P,C\}}^{\left(del\right)}$ represents the decrease of P=O bond delocalization and the degree to which the Lewis acid has weakened the P=O bond. In this sense, Δδ_33_ can be understood as an experimental spectroscopic approximation of the intrinsic P=O bond strength, with both quantities independently tracking the same underlying electronic consequences. Thus, one practical outcome of this study is that DFT-calculated energies are not strictly necessary to assess Lewis acid strength via the Gutmann–Beckett method—the ^31^P NMR chemical shift tensor, and in particular Δδ_33_ component, provides direct experimental access to the same information. This statement comes with the caveat that TEPO must bind sufficiently strongly to form a stable adduct and that measurements must be conducted in the absence of exchange dynamics.

## Conclusion

The experimentalist should exercise caution when using the Gutmann–Beckett method. This study shows that there are correlations between ^31^P NMR properties and P=O bond weakening, which is a thermodynamic descriptor of Lewis acid strength, but the correlation between the most commonly reported change in isotropic ^31^P NMR chemical shift (Δδ_iso_) and P=O bond strength is weak. Two solutions are proposed. Acquisition of solid-state ^31^P­{^1^H} NMR data to extract Δδ_33_ values that are correlated well with P=O bond weakening. In the absence of these measurements, solution *T*_1_ measurements at multiple field strengths allows the user to approximate $\Delta E_{O\to \{P,C\}}^{\left(del\right)}$ from the linear regression shown in Figure 9.

The reason for this behavior is that only the most shielded δ_33_ component of the ^31^P NMR chemical shift tensor is dramatically affected in TEPO adducts, and the changes in δ_33_ track with overall change in ^31^P NMR chemical shift far better than the more deshielded δ_11_ or δ_22_ components. NCS analysis shows that this is due to Δδ_33_ coupling filled P–C bonding orbitals to empty π* orbitals. Bond weakening narrows the π/π* HOMO–LUMO gap, which results in more paramagnetic deshielding as stronger Lewis acids further weaken the P=O bond.

This trend holds across a chemically diverse set of molecular Lewis acids and small cluster models that approximate Lewis sites on heterogeneous surfaces. The latter case is particularly valuable for those who study heterogeneous catalysts because Lewis sites play a significant role in activating substrates on these materials and their molecular structure is often difficult to assess or compare to small molecules.

Finally, Lewis acid strength is a nebulous parameter. Brønsted (H^+^) acidity is described by straightforward thermodynamic descriptors (e.g., p*K*_a_, deprotonation energy (DPE), etc.) because the proton does not need to structurally adapt to a base in the way that a Lewis acid must. This is part of the reason why so many of the useful scales that measure Lewis acidity (^19^F NMR,^52^ Fluoride ion affinity,^53^ Hydride ion affinity,^54^ Global electrophilicity index,^55^ etc.) usually do not correlate with one another. We do not propose a solution to this challenge, but hope that this study shows that the Gutmann–Beckett method, if viewed in more granular detail than just Δδ_iso_, has validity because P=O bond weakening is a thermodynamic descriptor of Lewis acid strength.

## Supplementary Material







## Data Availability

All underlying data sets presented and discussed in this study are available via 4TU.ResearchData at DOI: https://doi.org/10.4121/426e8bc2-27a5-4eb6-8fec-541c02733dd5.

## Notes

The geometries of the models under consideration (ZIP)
Raw data (CSV)
List of models, computational and experimental details, analysis of the principal components of the chemical shielding (PDF)
