# Supplementary material for: Origin of the 31P NMR Chemical Shift in Lewis Acid Adducts of Triethylphosphine Oxide. Does the Gutmann–Beckett Method Relate to Lewis Acid Strength?
Source: J Am Chem Soc. 2026 Apr 1;148(14):14768–78. doi: 10.1021/jacs.5c17621 (PMC13088190; doi:10.1021/jacs.5c17621)
Supplement: Supplementary file 3 [file ja5c17621_si_003.pdf]

SUPPLEMENTARY INFORMATION

FOR

# Origin of the $^{31}$P NMR Chemical Shift in Lewis Acid Adducts of Triethylphosphine Oxide. Does the Gutmann-Beckett Method Relate to Lewis Acid Strength?

Alexander A. Kolganov,[1] Maximillian Kling,[2] Matthew P. Conley,[2]* Evgeny A. Pidko[1]*

[1]*Inorganic Systems Engineering, Department of Chemical Engineering, Faculty of Applied Sciences, Delft University of Technology, Van der Maasweg 9, Delft, 2629 HZ, The Netherlands*

[2]*Department of Chemistry, University of California, Riverside, California 92507, United States*

*E-mails:* matthew.conley@ucr.edu *(M.P.C.);* e.a.pidko@tudelft.nl *(E.A.P.)*

# Section S1. General Considerations.

## S1.1 Solid State NMR Measurements.

TEPO was purchased from Sigma Aldrich and purified by sublimation prior to use for solid-state NMR measurements. TEPO-$B(C_6F_5)_3$ was prepared by a literature method using sublimed TEPO.[1] Solid state $^{31}P\{^1H\}$ NMR spectra were recorded in 4 mm zirconia rotors packed inside an inert atmosphere glovebox sealed with Kel-F caps. Spectra were acquired on a Bruker Neo-600 NMR spectrometer using a standard zg pulse sequence (π/2–acq) with a 3 s recycle delay between scans. All analytical simulations of solid-state spectra were performed in Topspin using Sola lineshape analysis.

## S1.2 Models.

The list of the molecular Lewis acids considered in this paper can be found in Table S1.1

**Table S1.1** Molecular Lewis acids considered in the manuscript

| Acid | 2D geom. | 3D geom. | Acid | 2D geom. | 3D geom. |
|---|---|---|---|---|---|
| $El(C_6F_5)_3$<br><br>El = B, Al | 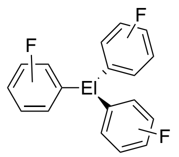 | 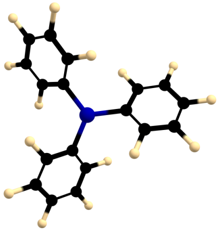 | $El(C_6H_5)_3$<br>El = B, Al | 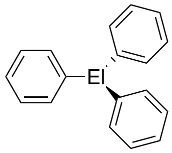 | 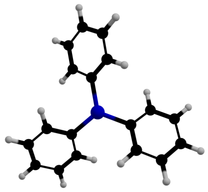 |
| Group 13 $ElX_3$<br>If El = **B**, **Al**, **Ga** then<br>X =F, Br, Cl, I<br>If El = **In**, then X = Cl | 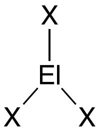 | 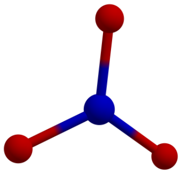 | $In(OTf)_3$ | 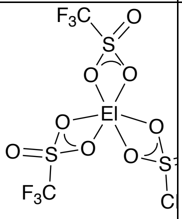 | 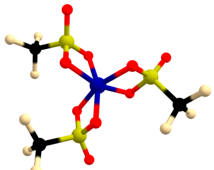 |
| $BiX_3$ (X = Cl, I) | 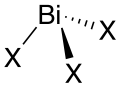 | 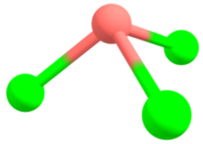 | $Bi(OTf)_3$ | 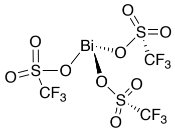 | 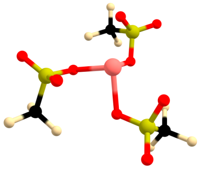 |
| $Al(OC(CF_3)_3)$ | 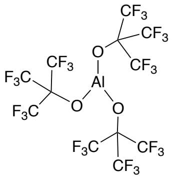 | 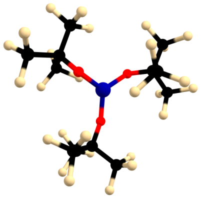 | $SO_3$ | 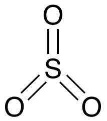 | 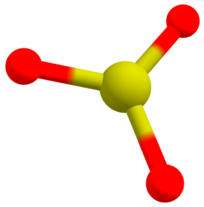 |
| $B(o\text{-}Cb)_3$<br><br>o-Cb = ortho-carboranyl | 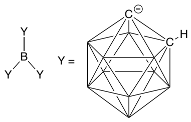 | 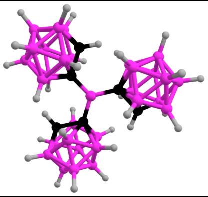 | H-B(2-Me-oCb)$_2$ | 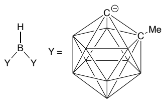 | 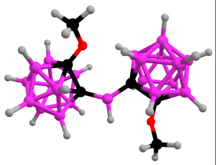 |

| | | | | | |
|---|---|---|---|---|---|
| Group 14 **$ElX_4$**<br>If El = **Si** or **Ge**, then X = F, Br, Cl<br>If El = **Sn**, then X = Cl | 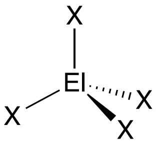 | 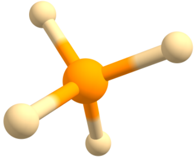 | Group 15 $ElX_5$<br>If El = As<br>X = F<br>If El = Sb:<br>X = F, Cl | 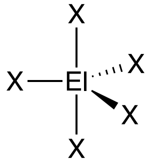 | 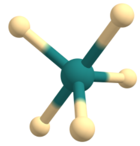 |
| Group 4 $ElX_4$<br>If El = Ti, then X = Cl, I<br>If El = Zr, then X = Cl | 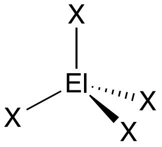 | 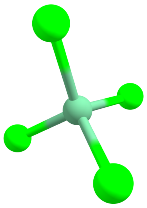 | Group 5 $ElCl_5$<br>El = Nb, Ta | 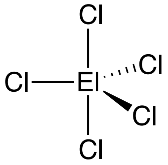 | 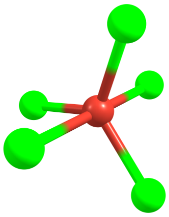 |
| $WF_6$ | 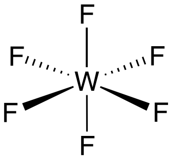 | 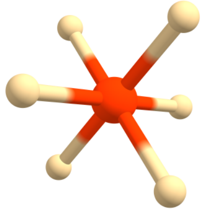 | $[(CH_3)_3C]^+$ | 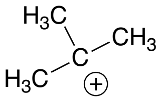 | 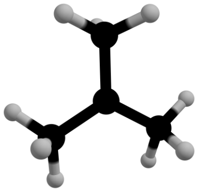 |
| $[Ph_3C]^+$ | 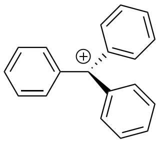 | 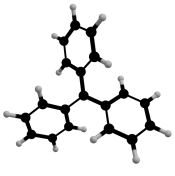 | $[Ge(C_6F_5)_3]^+$ | 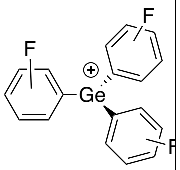 | 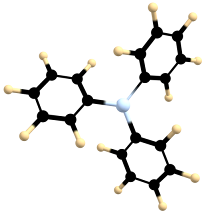 |
| $[SnPh_3]^+$ | 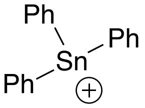 | 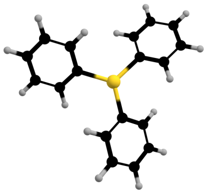 | $[Et_3Si]^+$ | 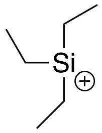 | 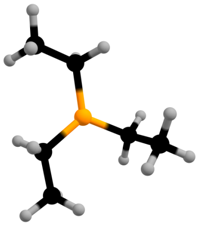 |
| $[BMes_2]^+$ | 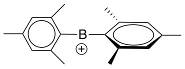 | 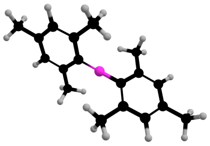 | $[Sb(C_6F_5)_4]^+$ | 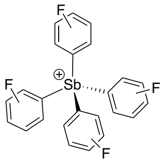 | 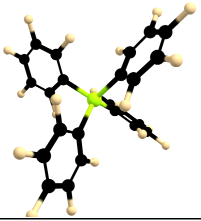 |

For the acids grafted on the amorphous silica, the support is represented via $(HO)_3SiO$- cluster, following the approach in the study Halbert et al.[2]

**Table S1.2** Silica-supported Lewis acids

| | | | | | |
|---|---|---|---|---|---|
| $[Al(OC(CF_3)_2)]@SiO_2$ | 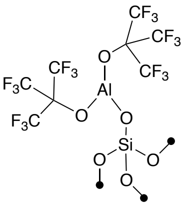 | 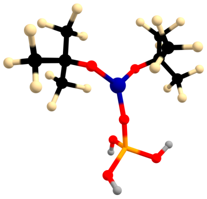 | [B(2-Me-oCb)$_2$]@$SiO_2$ | 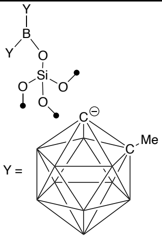 | 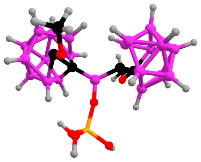 |

The cations confined in BEA zeolite are represented as 26T-atom cluster cut from the periodic zeolite framework as in the paper of Kolganov et al.[3] (Dangling oxygens were substituted by the hydrogen atoms and were fixed during geometry optimization to prevent unrealistic distortions of the zeolite framework. The models were named after the cation and T-atom positions of the Al atoms.

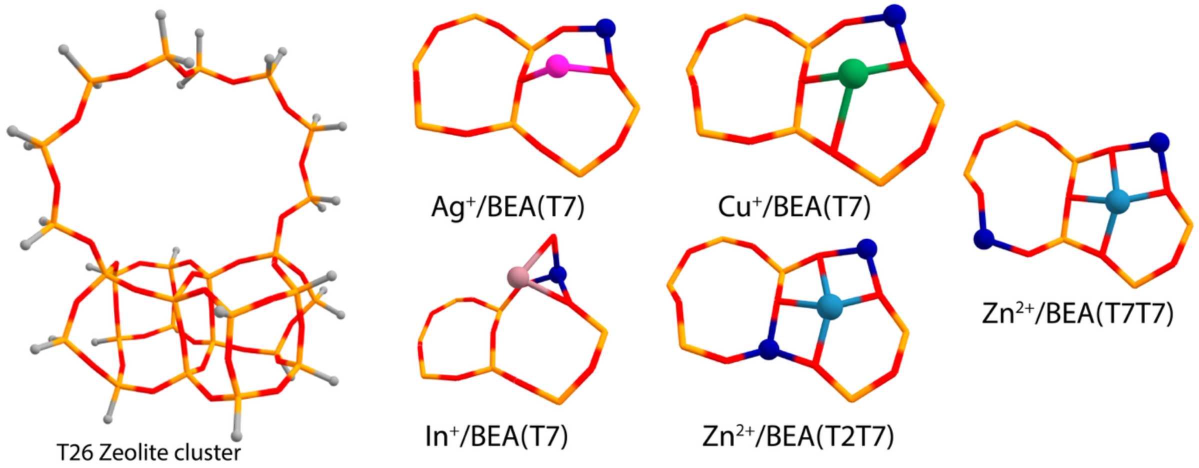


**Figure S1.1** Zeolite models used in this work. Al atoms are highlighted by navy blue.

To model sulfated zirconia (SZO) containing a pyrosulfate[4] we used a well-known $Zr_6O_4(OH)_4(HCOO)_{12}$ cluster[5] capped with acetate groups. One acetate was removed to accommodate the pyrosulfate group on top. The model is shown in Figure S1.2

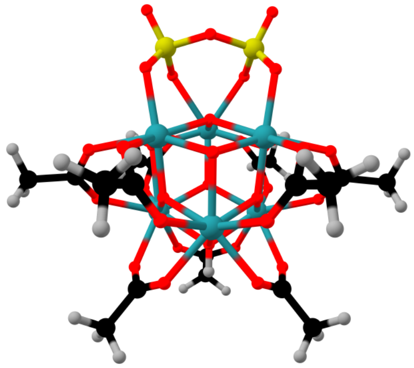

**Figure S1.2** Sulfated zirconia mimic (SZO) model. Color code: H (grey), C (black), O (red), S (yellow), Zr (blue)

## Section S2. Computational details.

**S2.1. Geometry optimization** was carried out in two steps. First, a conformer search was performed using CREST software[6] at the GFN2-XTB theory level.[7] From the CREST output, the most stable conformer has been selected for further DFT calculation if the run was successful. The geometries were optimized at ωB97M-D3BJ/def2-TZVPP[8] level of theory in ORCA 6.0 software package.[9]

**S2.2. Relativistic $^{31}$P NMR chemical shielding tensor calculations** of the adducts were calculated with Dirac-Kohn-Sham DFT using the relativistic four-component Dirac-Coulomb Hamiltonian (4c-DKS) as implemented in the ReSpect software.[10] The exchange-correlation energy was represented by the PBE0 functional.[11] Basis sets were assigned in the following way: for the atoms of TEPO and for acceptor atom in the acid, uncontracted pcS-2 was applied.[12] For the remaining atoms, we used uncontracted pcS-0 basis set.[13] If any of the atom is heavier than Kr ($Z>36$), we applied Dyall triple-zeta basis set (dyall-ctz).[14]

For TEPO adducts confined in zeolites, cluster models containing 12–13 T atoms around the active site were constructed (Figure S2.1) and capped with hydrogens placed along dangling Si–O bonds.

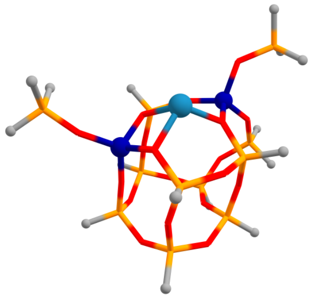

**Figure S2.1** Example of the smaller zeolite cluster used to calculate chemical shifts. TEPO molecule is omitted for clarity. Color code: Si (orange), O (red), Al (navy blue), Zn (cyan).

To reduce the calculation cost for the SZO model, methyl groups on capping acetates were substituted by H atoms.

**S2.3. Molecular orbital analysis** was carried out in the NBO 7.0[15] program interfaced with ORCA. The single point calculation was performed with the exact 2-component (X2C) Hamiltonian at PBE0-D3BJ/x2c-TZVPPall level of theory to match the method used to calculate $^{31}$P shielding constants.[16]

**S2.4. Natural chemical shift analysis (NCS)**[17] was carried out in the NBO 7.0[15] program interfaced with ORCA. For this analysis, chemical shielding was calculated using zero-order regular approximation for relativistic effects (ZORA).[18] Here, we used the same basis/functional as we used for the full relativistic NMR chemical shielding tensor calculations, except here for the heavy atoms SARC-ZORA-TZVPP basis set was used.[19]

**S2.5. Solid-State NMR** of the molecular TEPO crystal was calculated using CP2K 2024.3[20] and CASTEP 23.1.[21] The geometries were optimized in CP2K using Gaussian Plane Waves method

with PBE0 functional and revised Vydrov-van Voorhis nonlocal van der Waals density functional (rVV10) to account for non-covalent interactions.[22] DZVP-MOLOPT-SR-GTH basis set was used for the localized Gaussian part, while the 450/50 Ry cutoff/rel. cutoff were applied for plane waves. NMR parameters were calculated using the gauge-including projector augmented wave (GIPAW) method in CASTEP, [23] with a PBE functional employing a plane-wave cutoff of 600 eV. Calculated solid-state chemical shifts were referenced to the averaged over unit cell isotropic $^{31}P$ NMR chemical shielding tensor of $PPh_3$ molecular crystal.

**Table S2.1** Calculated chemical shielding tensor values for the $PPh_3$ molecular crystal.

| Atom | $\sigma_{iso}$ | $\sigma_{11}$ | $\sigma_{22}$ | $\sigma_{33}$ |
|---|---|---|---|---|
| 1 | 299.99 | 312.55 | 302.59 | 284.84 |
| 2 | 300.66 | 316.90 | 303.51 | 281.55 |
| 3 | 307.82 | 286.80 | 307.10 | 329.56 |
| 4 | 301.78 | 286.64 | 299.63 | 319.07 |
| 5 | 300.00 | 312.55 | 302.61 | 284.85 |
| 6 | 300.62 | 316.86 | 303.45 | 281.54 |
| 7 | 307.79 | 286.78 | 307.05 | 329.54 |
| 8 | 301.77 | 286.65 | 299.59 | 319.06 |
| Average | 302.55 | 300.72 | 303.19 | 303.75 |

# Section S3. Validation of the calculation methods

The reference values were taken from Prof. Hans Reich's collection.[24] The list of reference molecules is presented in Table S3.1, and the parity plot is shown in Figure S3.1.

**Table S3.1.** Experimental $^{31}$P isotropic chemical shift and DFT-calculated chemical shield for the reference set

| Molecule | Experimental $\delta(^{31}P)$ | DFT-calculated $\sigma(^{31}P)$ | Molecule | Experimental $\delta(^{31}P)$ | DFT-calculated $\sigma(^{31}P)$ |
|---|---|---|---|---|---|
| $O{=}PEt_3$ | 48.3 | 295.661 | $O{=}PPh_3$ | 25.5 | 312.536 |
| $O{=}P(OMe)_3$ | 2 | 330.066 | $S{=}PEt_3$ | 54.5 | 289.246 |
| $PtBu_3$ | 63 | 282.149 | $O{=}PH(OH)_2$ | 5 | 321.936 |
| $PMe_3$ | -62 | 402.908 | $O{=}PMe_2OEt$ | 50.3 | 286.994 |
| $PPh_3$ | -6 | 340.786 | $Se{=}P(OMe)_3$ | 78.4 | 247.511 |
| $O{=}PMe_3$ | 36.2 | 314.589 | | | |

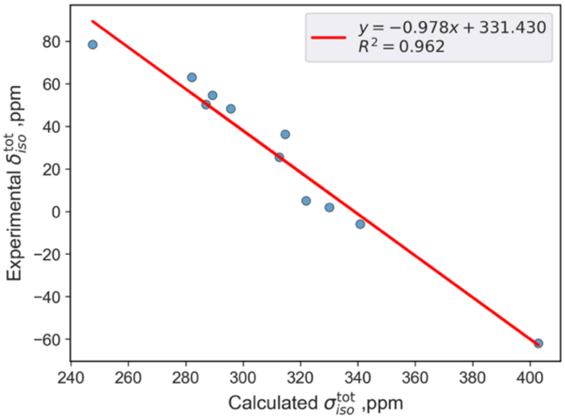


**Figure S3.1.** Calculated shielding constants of reference set vs. experimental data.

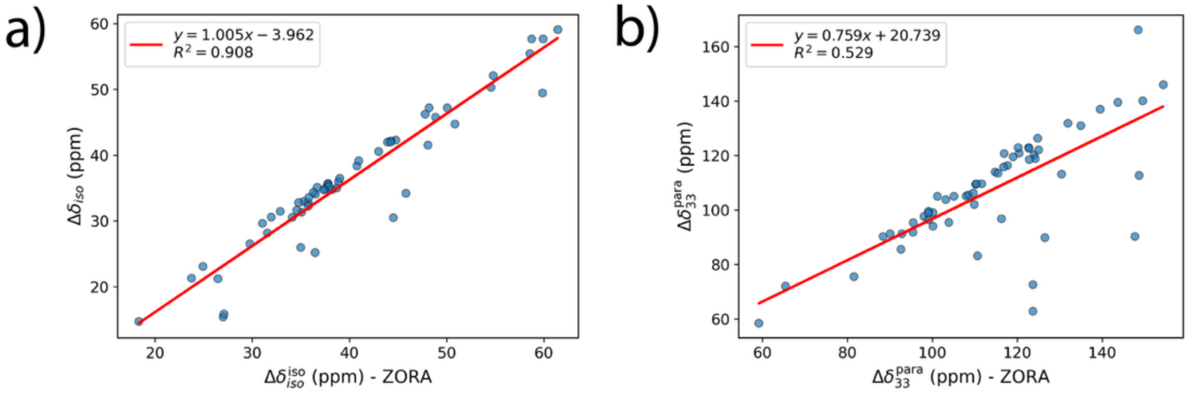


**Figure S3.2** Correlations between NMR parameters calculated with full 4-component Hamiltonian and ZORA Hamiltonian: change in isotropic chemical shift (a) change in 33 component of paramagnetic chemical shift (b)

# Section S4. Correlation of the Lewis acidity descriptors with the DFT-calculated gas-phase thermochemistry

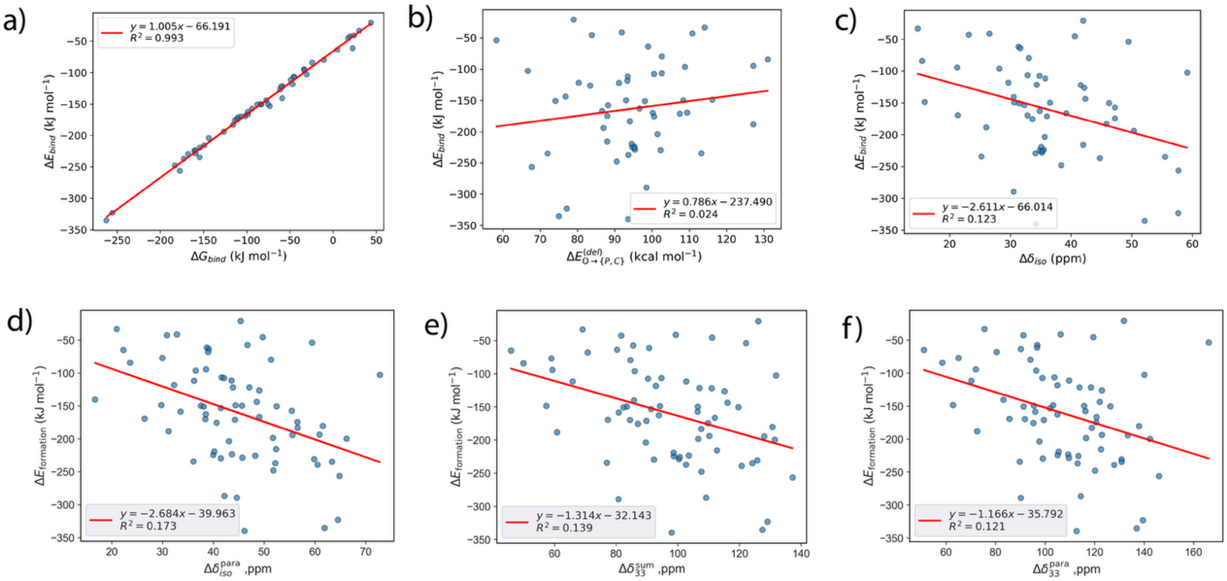


**Figure S4.1** (a) Correlation between the electronic adduct-formation energy, ΔE, and the corresponding gas-phase formation Gibbs free energy at 298 K, ΔG, for the TEPO+LA data set. The near-linear relationship ($R^2$ = 0.99) justifies the use of the electronic energy as the descriptor of Lewis-acid binding strength in the main text. (b) Plot of $\Delta E^{(del)}_{O\to\{P,C\}}$ vs $\Delta E_{bind}$. Plots of $\Delta E_{bind}$ versus NMR chemical shift components: the isotropic chemical shift change ($\Delta\delta_{iso}$, c), the isotropic paramagnetic shielding contribution ($\Delta\delta^{para}_{iso}$, d) the change in the total ($\Delta\delta_{33}$, e) and paramagnetic ($\Delta\delta^{para}_{33}$, f) for the most shielded component of the chemical shift tensor. There is no correlation between $\Delta E^{bind}$ and the NMR chemical shift components shown here.

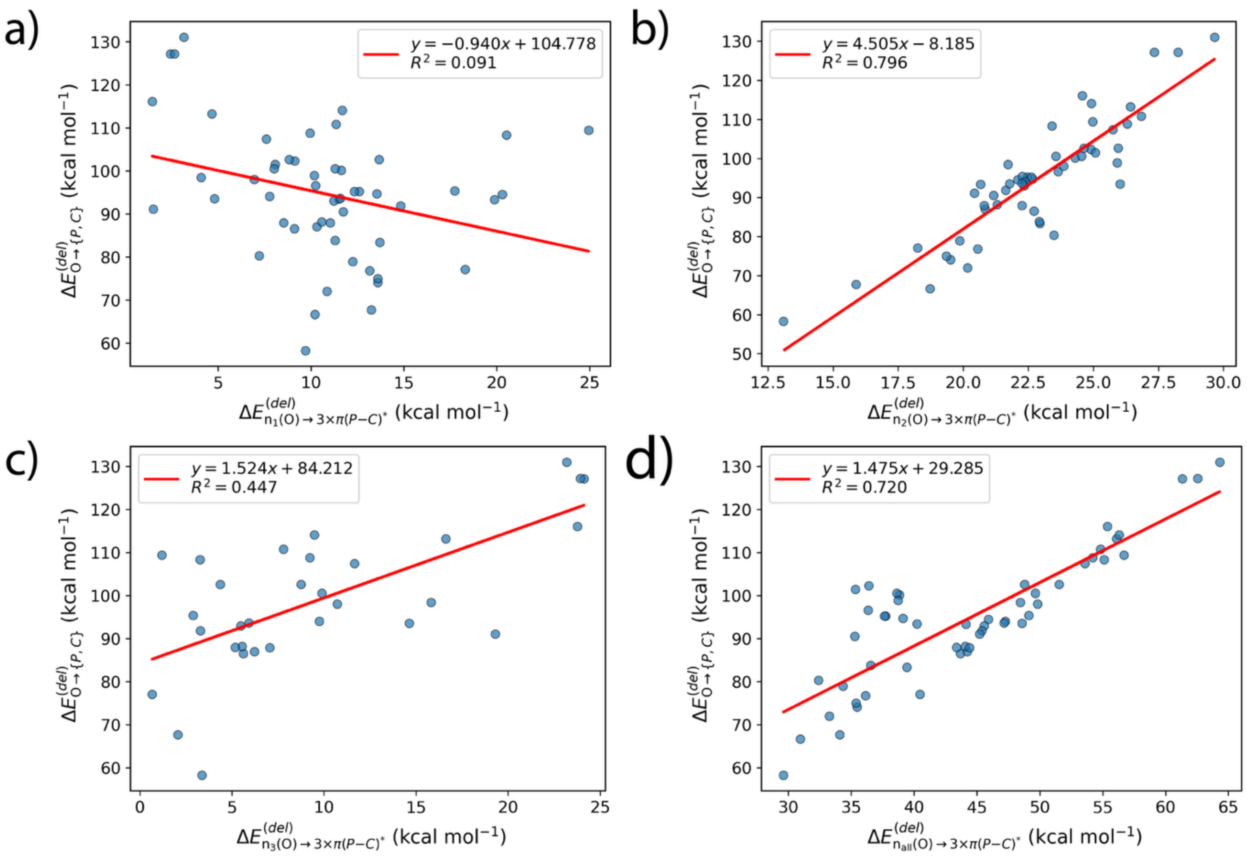


**Figure S4.2** Correlations between the individual (a-c)/combined (d) lone pair deletion energies and $\Delta E^{(del)}_{O\rightarrow\{P,C\}}$.

# Section S5. Analysis of the chemical shielding tensor components from DFT calculations.

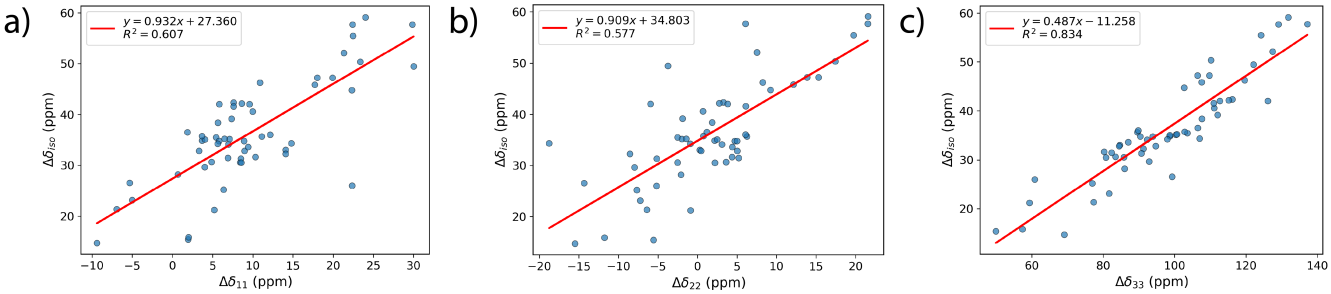


**Figure S5.1.** Plots of $\Delta\delta_{ii}$ versus $\Delta\delta_{iso}$.

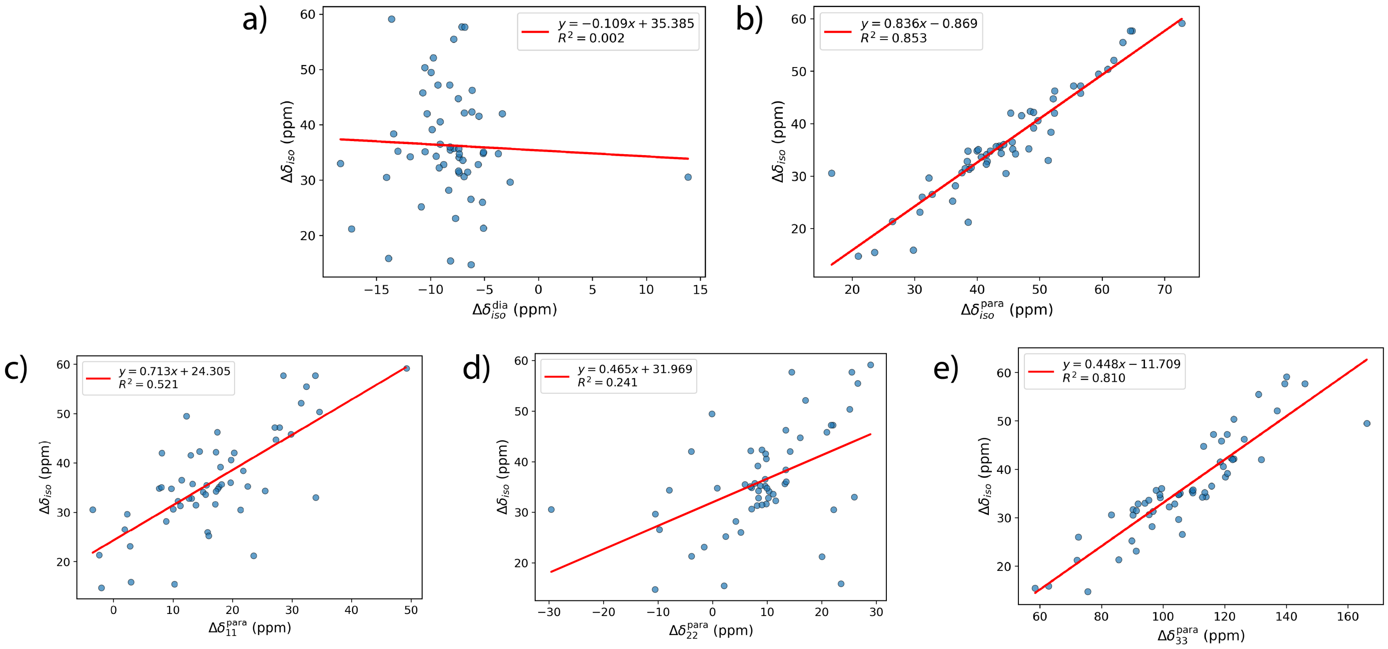


**Figure S5.2.** Plots of $\Delta\delta_{iso}$ versus $\Delta\delta_{iso}^{dia}$ (a) or $\Delta\delta_{iso}^{para}$ (b). Plots of $\Delta\delta_{iso}$ versus $\Delta\delta_{11}^{para}$ (c), $\Delta\delta_{22}^{para}$ (d), or $\Delta\delta_{33}^{para}$ (e).

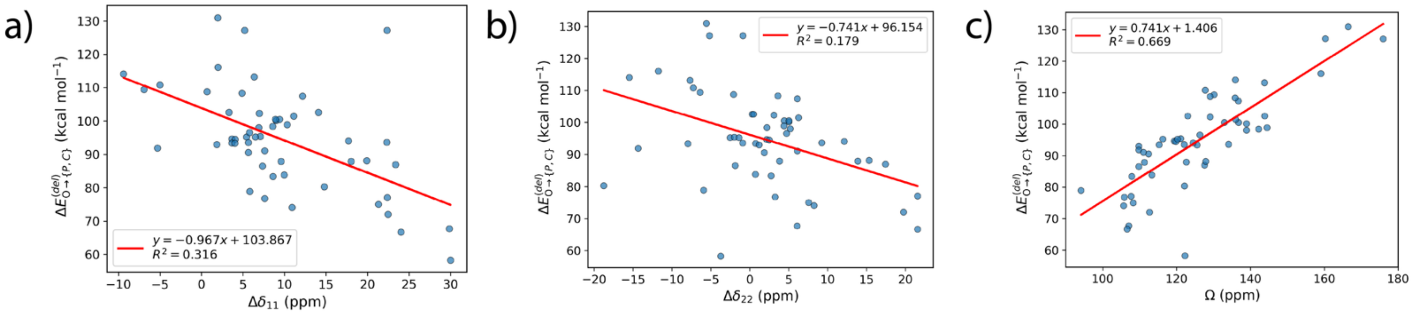


**Figure S5.3** (a) Plots of $\Delta\delta_{11}$ versus $\Delta E^{(del)}_{O\rightarrow\{P,C\}}$, (b) $\Delta\delta_{22}$ versus $\Delta E^{(del)}_{O\rightarrow\{P,C\}}$, (c) $\Omega$ versus $\Delta E^{(del)}_{O\rightarrow\{P,C\}}$

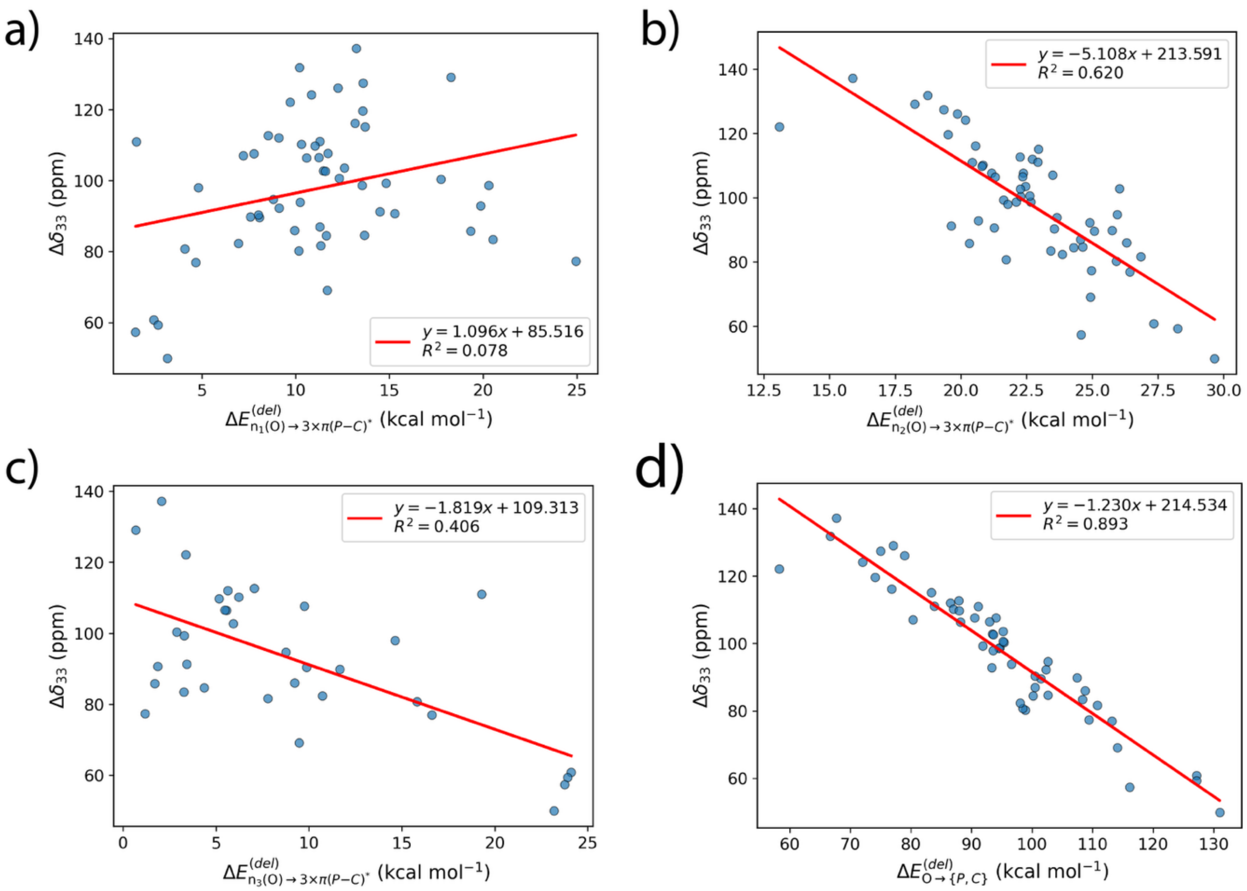


**Figure S5.4** (a) Plots of (a) $\Delta E^{(del)}_{n_1(O)\to 3\times\sigma*(P-C)}$, (b) $\Delta E^{(del)}_{n_2(O)\to 3\times\sigma*(P-C)}$ , (c) $\Delta E^{(del)}_{n_3(O)\to 3\times\sigma*(P-C)}$ (d) $\Delta E^{(del)}_{O\to\{P,C\}}$ vs. $\Delta\delta_{33}$

# Section S6 NLMOs $Cu^+$/BEA, $In^+$/BEA, and $Ag^+$/BEA.

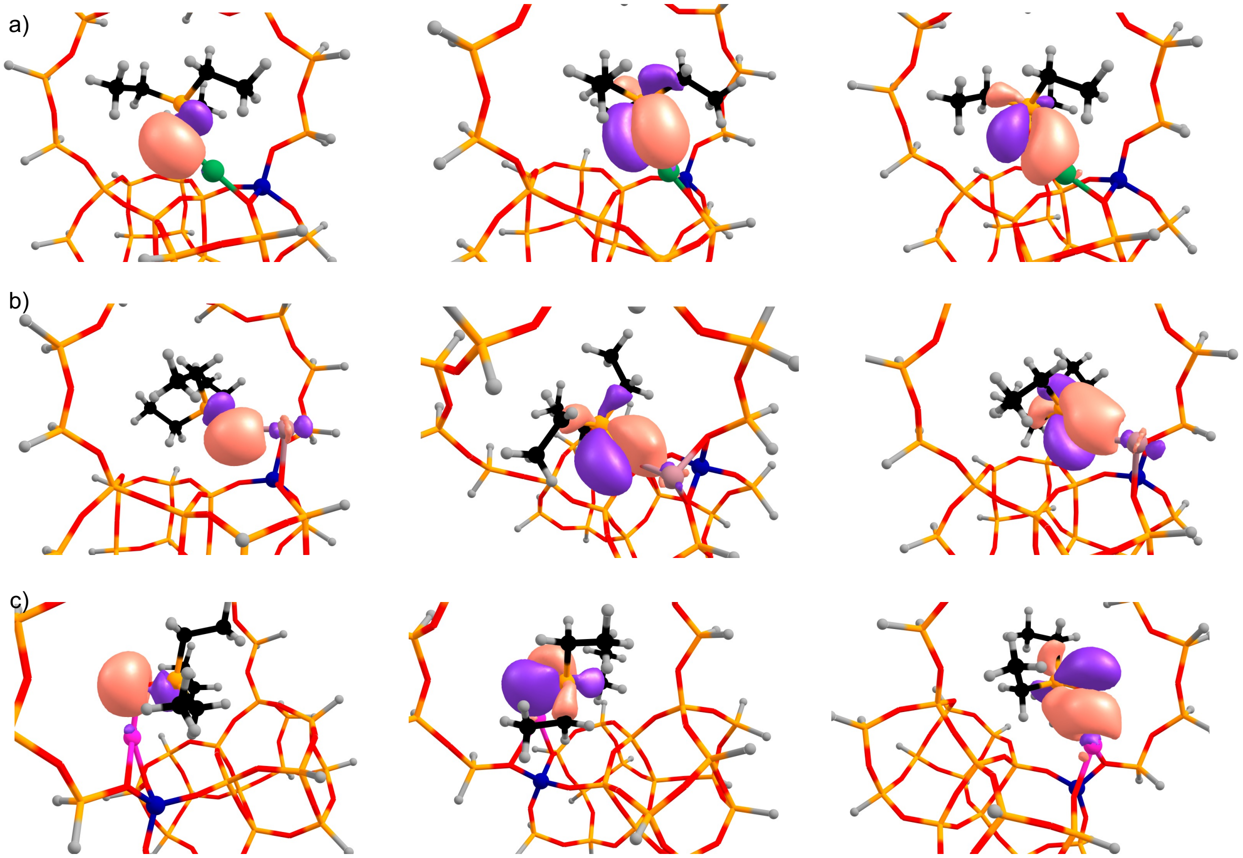


**Figure S6.1.** NLMOs of TEPO adducts for $Cu^+$/BEA (top), $In^+$/BEA (middle), and $Ag^+$/BEA (bottom). The $n_1$(O) orbital is shown on the left, $n_2$(O) is shown in the middle, and $n_3$(O) is shown on the right.

# Section S7. Raw data

**Table S7.1** Summary of ΔE, ∠E–O–P, $\Delta E_{O\rightarrow\{P,C\}}^{(del)}$, $\Delta\delta_{iso}$, and chemical shift tensor values for TEPO adducts of Lewis acids. The chemical shift tensor values are calculated using 4c-DKS Hamiltonian as described above. All energies are listed in kcal/mol

| Lewis Acid | ΔE | ∠E–O–P (°) | $\Delta E_{n_x(O)\rightarrow 3\times\sigma*(P-C)}^{(del)}$ | | | | $\Delta E_{O\rightarrow\{P,C\}}^{(del)}$ | $\Delta\delta_{iso}$ (exp) | $\Delta\delta_{iso}$ (DFT) | $\Delta\delta_{11}$ | $\Delta\delta_{22}$ | $\Delta\delta_{33}$ |
|---|---|---|---|---|---|---|---|---|---|---|---|---|
| | | | 1 | 2 | 3 | All | | | | | | |
| - | - | - | 2.01 | 38.1 | 37.5 | | 182.2 | | See text | | | |
| $B(C_6F_5)_3$ | –28.2 | 156.1 | 19.86 | 20.66 | | 44.12 | 93.354 | 26.5[a] | 29.6 | 4.0 | -8.0 | 92.9 |
| $BPh_3$ | –19.0 | 129.9 | 13.66 | 24.63 | 4.35 | 48.79 | 102.615 | 26.8[25] | 33.0 | 14.1 | 0.3 | 84.6 |
| $Al(C_6F_5)_3$ | –54.6 | 160.9 | 17.74 | 22.27 | 2.88 | 49.12 | 95.391 | 29.8[26] | 35.2 | 7.1 | –2.0 | 98.6 |
| $AlPh_3$ | –40.5 | 179.8 | 24.94 | 24.97 | 1.18 | 56.69 | 109.413 | - | 21.3 | –6.9 | –6.4 | 77.3 |
| $BF_3$ | –26.7 | 124.1 | 11.48 | 26.03 | | 40.24 | 93.462 | 29.0[25] | 35.7 | 3.7 | 0.8 | 102.8 |
| $BCl_3$ | –30.2 | 133.8 | 13.69 | 22.94 | | 39.42 | 83.388 | 38.7[25] | 42.2 | 8.6 | 2.7 | 115.1 |
| $BBr_3$ | –34.3 | 132.6 | 13.16 | 20.54 | | 36.1 | 76.796 | 40.3[25] | 42.3 | 7.6 | 3.3 | 116.1 |
| $BI_3$ | –36.0 | 136.2 | 13.5 | 19.51 | | 35.42 | 74.093 | 42.9[25] | 46.3 | 10.9 | 8.2 | 119.6 |
| $AlF_3$ | –54.8 | 134.2 | 9.11 | 24.90 | | 36.39 | 102.293 | - | 34.1 | 7.0 | 3.1 | 92.3 |
| $AlCl_3$ | –53.4 | 140.5 | 12.59 | 22.45 | | 37.74 | 95.228 | 36.0[26] | 35.5 | 5.4 | –2.5 | 103.6 |
| $AlBr_3$ | –53.6 | 147.7 | 13.5 | 22.65 | | 39.11 | 94.681 | 35.4[27] | 34.8 | 3.7 | 2.2 | 98.7 |
| $AlI_3$ | –52.4 | 151.8 | 20.29 | 22.1 | | 45.89 | 94.502 | 37.4[27] | 35.1 | 4.1 | 2.5 | 98.6 |
| $GaF_3$ | –48.7 | 132.1 | 8.07 | 25.07 | | 35.34 | 101.475 | - | 35.7 | 11.1 | 6.3 | 89.6 |
| $GaCl_3$ | –42.0 | 141.1 | 11.29 | 24.54 | | 38.63 | 100.519 | 31.1[26] | 33.6 | 9.4 | 4.4 | 87.0 |
| $GaBr_3$ | –40.6 | 143.7 | 11.62 | 24.29 | | 38.83 | 100.162 | 21.0[28] | 32.8 | 9.0 | 5.0 | 84.5 |
| $GaI_3$ | –38.9 | 136.4 | 10.2 | 23.65 | | 36.34 | 96.602 | 30.2[27] | 34.8 | 5.8 | 4.7 | 93.9 |
| $InCl_3$ | –40.9 | 134.3 | 7.59 | 25.75 | 11.66 | 53.57 | 107.407 | 13.7[27] | 36.0 | 12.2 | 6.1 | 89.8 |
| $In(OTf)_3$ | –43.8 | 132.7 | 7.78 | 22.37 | 9.74 | 47.25 | 94.063 | - | 45.8 | 17.7 | 12.1 | 107.6 |
| $BiCl_3$ | –22.6 | 137.4 | 2.66 | 28.25 | 23.92 | 62.55 | 127.18 | 25.9[27] | 21.2 | 5.2 | –0.9 | 59.3 |
| $BiI_3$ | –20.2 | 143.9 | 3.16 | 29.65 | 23.18 | 64.31 | 130.995 | - | 15.4 | 2.0 | –5.6 | 49.9 |
| $Bi(OTf)_3$ | –46.3 | 151.4 | 10.31 | 20.83 | 6.21 | 44.21 | 86.996 | - | 50.3 | 23.4 | 17.4 | 110.2 |
| $Al(OR^F)_3$ | –59.2 | 147.2 | 11.73 | 21.16 | | 35.29 | 90.549 | - | 38.4 | 5.6 | 1.9 | 107.7 |
| $SO_3$ | –29.1 | 118.9 | 7.20 | 23.49 | | 32.39 | 80.316 | - | 34.3 | 14.8 | –18.8 | 107.0 |
| B(*o*-Cb)$_3$ | –33.6 | 177.1 | 19.32 | 20.32 | 1.70 | 53.86 | c | 30.1[29] | 30.6 | 8.4 | –2.5 | 85.8 |
| HB(*o*-Me-Cb) | –36.6 | 140.5 | 14.49 | 19.62 | 3.41 | 50.77 | c | 30.0[30] | 32.3 | 14.1 | –8.6 | 91.3 |

| | | | | | | | | | | | | |
|---|---|---|---|---|---|---|---|---|---|---|---|---|
| $SiF_4$ | –10.9 | 128.6 | 11.29 | 22.92 | | 36.54 | 83.84 | - | 40.6 | 10.0 | 0.7 | 111.1 |
| $SiCl_4$ | –5.0 | 137.2 | 12.25 | 19.86 | | 34.36 | 78.939 | - | 42.0 | 5.8 | –5.9 | 126.1 |
| $SiBr_4$ | –9.8 | 146.0 | 14.81 | 21.62 | 3.28 | 45.38 | 91.859 | - | 26.5 | –5.3 | –14.3 | 99.3 |
| $GeF_4$ | –25.4 | 132.1 | 8.82 | 25.94 | 8.76 | 51.54 | 102.605 | - | 32.8 | 3.3 | 0.5 | 94.7 |
| $GeCl_4$ | –10.2 | 145.3 | 11.35 | 26.84 | 7.79 | 54.83 | 110.801 | 21.5[31] | 23.1 | –5.0 | –7.3 | 81.7 |
| $GeBr_4$ | –8.0 | 153.8 | 11.68 | 24.92 | 9.47 | 56.30 | 114.073 | - | 14.8 | –9.4 | –15.5 | 69.1 |
| $SnCl_4$ | –23.0 | 145.2 | 9.95 | 26.30 | 9.23 | 54.2 | 108.779 | - | 28.2 | 0.7 | –2.1 | 86.0 |
| $AsF_5$ | –39.8 | 129.2 | 9.11 | 22.72 | 5.62 | 43.62 | 86.548 | - | 39.2 | 7.3 | –1.9 | 112.0 |
| $SbF_5$ | –51.5 | 130.5 | 8.53 | 22.25 | 7.04 | 44.40 | 87.927 | - | 42.0 | 9.6 | 3.8 | 112.7 |
| $SbCl_5$ | –35.8 | 141.7 | 11.23 | 22.35 | 5.46 | 45.57 | 92.999 | 36.1[1] | 36.5 | 1.9 | 1.2 | 106.5 |
| $TiCl_4$ | –25.7 | 143.0 | 8.02 | 23.55 | 9.88 | 49.63 | 100.521 | 22.7[1] | 34.8 | 8.9 | 5.0 | 90.4 |
| $TiI_4$ | –29.1 | 177.3 | 1.50 | 20.42 | 19.29 | 45.21 | 91.118 | - | 41.6 | 7.6 | 6.1 | 111.0 |
| $ZrCl_4$ | –36.1 | 149.5 | 6.95 | 23.86 | 10.71 | 49.83 | 98.037 | - | 31.5 | 6.9 | 5.2 | 82.3 |
| $NbCl_5$ | –37.6 | 147.1 | 10.58 | 21.30 | 5.55 | 44.07 | 88.17 | - | 47.2 | 19.9 | 15.3 | 106.4 |
| $TaCl_5$ | –41.7 | 147.6 | 11.03 | 20.79 | 5.1 | 43.37 | 87.943 | - | 47.2 | 18.0 | 13.9 | 109.8 |
| $WF_6$ | –15.2 | 134.6 | 10.16 | 25.91 | | 38.72 | 98.901 | - | 31.7 | 10.3 | 4.4 | 80.3 |
| $[C(CH_3)_3]^+$ | –56.1 | 133.2 | 10.85 | 20.17 | | 33.25 | 72.005 | - | 55.5 | 22.5 | 19.7 | 124.2 |
| $[Ph_3C]^+$ | –24.5 | 132.1 | 10.20 | 18.77 | | 30.96 | 66.685 | - | 59.1 | 24.0 | 21.6 | 131.8 |
| $[Ge(C_6F_5)_3]^+$ | –77.2 | 175.2 | 18.29 | 18.24 | 0.66 | 40.47 | 77.105 | - | 57.7 | 23.4 | 21.6 | 129.1 |
| $[Ph_3Sn]^+$ | –56.7 | 144.4 | 11.58 | 22.26 | 5.92 | 47.15 | 93.642 | - | 44.7 | 22.3 | 9.2 | 102.7 |
| $[Et_3Si]^+$ | -80.1 | 144.1 | 13.59 | 19.35 | | 35.38 | 75.027 | 42.4[b,32] | 52.1 | 21.3 | 7.5 | 127.5 |
| $Sb(C_6F_5)_4^+$ | –35.8 | 178.0 | 20.51 | 23.41 | 3.26 | 55.11 | 108.342 | 23.6[33] | 30.7 | 4.9 | 3.6 | 83.4 |
| $[BMes]_2^+$ | –61.3 | 137.9 | 13.24 | 15.87 | 2.06 | 34.09 | 67.717 | - | 57.7 | 29.8 | 6.1 | 137.2 |
| $Al(OR^F)_3/SiO_2$ | –53.9 | 142.6 | 12.33 | 22.62 | | 37.64 | 95.23 | 28[a,34] | 35.2 | 6.5 | –1.4 | 100.6 |
| HB(*o*-Me-Cb)/$SiO_2$ | –14.7 | 145.0 | 15.28 | 21.26 | 1.86 | 50.88 | c | 28[a,35] | 31.3 | 8.5 | –5.2 | 90.7 |
| $Zn^{2+}$/BEA (T2T7) | –69.1 | 149.6 | 4.09 | 21.72 | 15.81 | 48.45 | 98.449 | - | 30.5 | 8.6 | 2.2 | 80.8 |
| $Zn^{2+}$/BEA (T7T7) | –81.2 | 135.9 | 4.81 | 21.78 | 14.63 | 48.58 | 93.563 | - | 34.2 | 5.6 | –0.9 | 98.0 |
| $Cu^+$/BEA (T7) | –56.0 | 125.3 | 4.67 | 26.42 | 16.61 | 56.08 | 113.203 | - | 25.2 | 6.4 | –7.7 | 77.0 |
| $Ag^+$/BEA (T7) | –45.0 | 110.8 | 2.43 | 27.34 | 24.11 | 61.33 | 127.152 | - | 26 | 22.4 | –5.2 | 60.8 |
| $S_2O_7^{2-}/ZrO_2$ | –12.8 | 131.2 | 9.70 | 13.09 | 3.37 | 29.61 | 58.273 | 46[a,36] | 49.5 | 30.0 | –3.8 | 122.1 |
| $In^+$/BEA (T7) | –35.5 | 136.0 | 1.46 | 24.57 | 23.76 | 55.38 | 116.108 | - | 15.9 | 2.0 | –11.8 | 57.4 |

[a] – measured in the solid-state; [b] – anion = $[B(C_6F_5)_4]$; [c] – $\Delta E^{(del)}_{O\rightarrow\{P,C\}}$ calculations complicated by further delocalization effects from the carborane substitutents, thus these were omitted from the dataset.

**Table S7.2** Chemical shielding data for molecular TEPO

| $\sigma_{11}^{tot}$ | $\sigma_{22}^{tot}$ | $\sigma_{33}^{tot}$ | $\sigma_{11}^{dia}$ | $\sigma_{22}^{dia}$ | $\sigma_{33}^{dia}$ | $\sigma_{iso}^{dia}$ | $\sigma_{11}^{para}$ | $\sigma_{22}^{para}$ | $\sigma_{33}^{para}$ | $\sigma_{iso}^{para}$ | $\sigma_{iso}^{tot}$ |
|---|---|---|---|---|---|---|---|---|---|---|---|
| 223.93 | 224.73 | 438.32 | 962.95 | 964.954 | 969.74 | 965.88 | -739.02 | -740.23 | -531.42 | -670.22 | 295.66 |

**Table S7.3** Chemical shielding tensor values for TEPO adducts of Lewis acids. The chemical shielding tensor values are calculated using 4c-DKS as described above

| Lewis Acid | $\Delta\delta_{iso}^{dia}$ | $\Delta\delta_{11}^{dia}$ | $\Delta\delta_{22}^{dia}$ | $\Delta\delta_{33}^{dia}$ | $\Delta\delta_{iso}^{para}$ | $\Delta\delta_{11}^{para}$ | $\Delta\delta_{22}^{para}$ | $\Delta\delta_{33}^{para}$ |
|---|---|---|---|---|---|---|---|---|
| $B(C_6F_5)_3$ | -2.618 | 1.722 | 2.561 | -12.139 | 32.262 | 2.3 | -10.521 | 105.009 |
| $BPh_3$ | -18.335 | -19.902 | -25.669 | -9.435 | 51.341 | 33.997 | 25.964 | 94.063 |
| $Al(C_6F_5)_3$ | -10.52 | -10.668 | -11.595 | -9.298 | 45.677 | 17.753 | 9.612 | 109.666 |
| $AlPh_3$ | -5.103 | -4.537 | -2.525 | -8.248 | 26.431 | -2.401 | -3.88 | 85.575 |
| $BF_3$ | -7.828 | -9.61 | -6.971 | -6.903 | 43.564 | 13.279 | 7.728 | 109.686 |
| $BCl_3$ | -6.864 | -8.603 | -4.243 | -7.748 | 49.033 | 17.215 | 6.989 | 122.897 |
| $BBr_3$ | -6.178 | -6.859 | -5.757 | -5.919 | 48.516 | 14.468 | 9.014 | 122.068 |
| $BI_3$ | -6.16 | -6.557 | -5.17 | -6.753 | 52.409 | 17.453 | 13.414 | 126.361 |
| $AlF_3$ | -7.368 | -8.097 | -7.217 | -6.792 | 41.479 | 15.047 | 10.32 | 99.071 |
| $AlCl_3$ | -8.167 | -10.206 | -8.467 | -5.829 | 43.649 | 15.626 | 5.928 | 109.394 |
| $AlBr_3$ | -5.129 | -3.989 | -5 | -6.398 | 39.966 | 7.663 | 7.156 | 105.082 |
| $AlI_3$ | -5.093 | -3.998 | -4.391 | -6.891 | 40.155 | 8.046 | 6.883 | 105.538 |
| $GaF_3$ | -7.387 | -7.057 | -6.98 | -8.124 | 43.036 | 18.177 | 13.238 | 97.695 |
| $GaCl_3$ | -7.022 | -6.067 | -6.691 | -8.31 | 40.632 | 15.484 | 11.098 | 95.316 |
| $GaBr_3$ | -5.581 | -4.133 | -5.195 | -7.416 | 38.405 | 13.112 | 10.221 | 91.882 |
| $GaI_3$ | -3.718 | -3.922 | 3.874 | -11.105 | 38.507 | 9.715 | 0.836 | 104.97 |
| $InCl_3$ | -8.2 | -7.509 | -7.416 | -9.675 | 44.221 | 19.665 | 13.507 | 99.493 |
| $In(OTf)_3$ | -10.728 | -12.111 | -8.8 | -11.272 | 56.542 | 29.81 | 20.941 | 118.876 |
| $BiCl_3$ | -17.327 | -18.34 | -20.913 | -12.728 | 38.541 | 23.53 | 20.038 | 72.055 |
| $BiI_3$ | -8.164 | -8.281 | -7.669 | -8.542 | 23.6 | 10.241 | 2.089 | 58.47 |
| $Bi(OTf)_3$ | -10.541 | -11.237 | -7.666 | -12.723 | 60.878 | 34.608 | 25.11 | 122.918 |
| $Al(OR^F)_3$ | -13.427 | -16.171 | -11.554 | -12.557 | 51.81 | 21.811 | 13.411 | 120.208 |
| $SO_3$ | -9.486 | -10.705 | -10.828 | -6.925 | 43.828 | 25.486 | -7.941 | 113.942 |
| B(*o*-Cb)$_3$ | 13.858 | 11.912 | 27.073 | 2.588 | 16.703 | -3.499 | -29.592 | 83.2 |
| HB(*o*-Me-Cb) | -9.2 | 3.258 | -20.125 | -10.734 | 41.467 | 10.824 | 11.569 | 102.01 |
| $SiF_4$ | -9.117 | -9.78 | -9.129 | -8.443 | 49.697 | 19.752 | 9.829 | 119.51 |
| $SiCl_4$ | -3.353 | -2.289 | -1.999 | -5.771 | 45.354 | 8.11 | -3.934 | 131.886 |
| $SiBr_4$ | -6.256 | -7.213 | -4.595 | -6.96 | 32.799 | 1.891 | -9.746 | 106.253 |

| | | | | | | | | |
|---|---|---|---|---|---|---|---|---|
| $GeF_4$ | -8.791 | -9.375 | -7.951 | -9.049 | 41.616 | 12.681 | 8.409 | 103.76 |
| $GeCl_4$ | -7.691 | -7.779 | -5.692 | -9.605 | 30.821 | 2.774 | -1.568 | 91.257 |
| $GeBr_4$ | -6.247 | -7.377 | -4.942 | -6.422 | 20.985 | -2.019 | -10.548 | 75.523 |
| $SnCl_4$ | -8.329 | -8.191 | -6.356 | -10.443 | 36.511 | 8.855 | 4.259 | 96.42 |
| $AsF_5$ | -9.855 | -10.62 | -10.141 | -8.806 | 49.021 | 17.971 | 8.268 | 120.825 |
| $SbF_5$ | -10.328 | -10.688 | -10.394 | -9.902 | 52.348 | 20.263 | 14.199 | 122.582 |
| $SbCl_5$ | -9.101 | -9.614 | -8.421 | -9.269 | 45.615 | 11.463 | 9.606 | 115.776 |
| $TiCl_4$ | -7.337 | -8.591 | -4.928 | -8.494 | 42.1 | 17.5 | 9.946 | 98.855 |
| $TiI_4$ | -5.517 | -5.368 | -3.59 | -7.595 | 47.075 | 12.969 | 9.689 | 118.566 |
| $ZrCl_4$ | -6.578 | -6.934 | -3.81 | -8.991 | 38.055 | 13.825 | 9.022 | 91.32 |
| $NbCl_5$ | -8.22 | -7.991 | -6.729 | -9.941 | 55.438 | 27.904 | 22.048 | 116.361 |
| $TaCl_5$ | -9.314 | -9.099 | -7.857 | -10.987 | 56.53 | 27.114 | 21.738 | 120.738 |
| $WF_6$ | -7.411 | -6.779 | -5.46 | -9.994 | 39.058 | 17.09 | 9.839 | 90.245 |
| $[C(CH_3)_3]^+$ | -7.86 | -9.946 | -6.872 | -6.761 | 63.328 | 32.415 | 26.616 | 130.955 |
| $[Ph_3C]^+$ | -13.616 | -25.213 | -7.378 | -8.258 | 72.752 | 49.24 | 28.94 | 140.078 |
| $[Ge(C_6F_5)_3]^+$ | -6.837 | -6.132 | -3.974 | -10.406 | 64.518 | 28.524 | 25.519 | 139.512 |
| $[Ph_3Sn]^+$ | -7.423 | -4.986 | -6.805 | -10.479 | 52.159 | 27.3 | 16.032 | 113.146 |
| $[Et_3Si]^+$ | -9.752 | -10.197 | -9.493 | -9.566 | 61.858 | 31.524 | 17.033 | 137.017 |
| $Sb(C_6F_5)_4^+$ | -6.896 | -5.125 | -3.541 | -12.025 | 37.532 | 10 | 7.151 | 95.445 |
| $[BMes]_2^+$ | -7.114 | -4.062 | -8.441 | -8.841 | 64.817 | 33.91 | 14.517 | 146.027 |
| $Al(OR^F)_3/SiO_2$ | -13.026 | -16.062 | -10.158 | -12.86 | 48.253 | 22.539 | 8.748 | 113.475 |
| HB(*o*-Me-Cb)/$SiO_2$ | -7.359 | -2.697 | -13.317 | -6.066 | 38.699 | 11.22 | 8.143 | 96.733 |
| $Zn^{2+}$/BEA (T2T7) | -14.08 | -12.75 | -19.999 | -9.491 | 44.591 | 21.329 | 22.194 | 90.248 |
| $Zn^{2+}$/BEA (T7T7) | -11.877 | -11.576 | -9.326 | -14.73 | 46.111 | 17.215 | 8.421 | 112.696 |
| $Cu^+$/BEA (T7) | -10.864 | -9.638 | -10.072 | -12.882 | 36.074 | 15.991 | 2.39 | 89.84 |
| $Ag^+$/BEA (T7) | -5.185 | 6.535 | -10.357 | -11.735 | 31.185 | 15.82 | 5.176 | 72.559 |
| $S_2O_7^{2-}/ZrO_2$ | -9.962 | 17.757 | -3.639 | -44.003 | 59.418 | 12.248 | -0.111 | 166.117 |
| $In^+$/BEA (T7) | -13.903 | -0.947 | -35.273 | -5.491 | 29.768 | 2.943 | 23.519 | 62.842 |

**Table S7.4.** NCS Analysis $\sigma_{11}^{para}$.

| Lewis Acid | $\sigma_{11}^{para}$ (P–O) | $\sigma_{11}^{para}$ (P–C) | $\sigma_{11}^{para}$ (P–C) | $\sigma_{11}^{para}$ (P–C) |
|---|---|---|---|---|
| - | -232 | -221 | -83 | -84 |
| $B(C_6F_5)_3$ | -166.23 | -284.81 | -169.51 | -297.36 |
| $BPh_3$ | -164.06 | -243.81 | -163.23 | -282.91 |
| $Al(C_6F_5)_3$ | -154.93 | -298.28 | -119.6 | -259.59 |
| $AlPh_3$ | -170.75 | -285.29 | -73.53 | -201.86 |
| $BF_3$ | -199.57 | -224.01 | -223.98 | -268.04 |
| $BCl_3$ | -186.91 | -241.25 | -241.04 | -288.14 |
| $BBr_3$ | -180.4 | -293.18 | -175.05 | -281.91 |
| $BI_3$ | -171.55 | -320.58 | -99.84 | -251.7 |
| $AlF_3$ | -186.22 | -276.52 | -139.22 | -249.25 |
| $AlCl_3$ | -178.14 | -290.02 | -105.79 | -231.31 |
| $AlBr_3$ | -180.51 | -297.39 | -116.3 | -243.47 |
| $AlI_3$ | -175.79 | -268.53 | -182.9 | -292.23 |
| $GaF_3$ | -186.46 | -279.66 | -143.48 | -254.55 |
| $GaCl_3$ | -183.83 | -292.27 | -123.99 | -249 |
| $GaBr_3$ | -160.29 | -294.38 | -123.35 | -250.07 |
| $GaI_3$ | -175.71 | -243.32 | -211.93 | -301.33 |
| $InCl_3$ | -184.55 | -224.04 | -222.91 | -282.5 |
| $In(OTf)_3$ | -161.06 | -296.87 | -103.97 | -241.85 |
| $BiCl_3$ | -199.35 | -262.85 | -134.2 | -247.36 |
| $BiI_3$ | -204.25 | -271.86 | -94.41 | -211.82 |
| $Bi(OTf)_3$ | -163.98 | -282.7 | -231.58 | -344.69 |
| $Al(OR^F)_3$ | -173.79 | -283.02 | -106.92 | -236.54 |
| $SO_3$ | -177.78 | -273.37 | -175.34 | -259.58 |
| B(*o*-Cb)$_3$ | -124.67 | -260.43 | -203.55 | -308.96 |
| HB(*o*-Me-Cb) | -139.47 | -281.31 | -97.8 | -229.32 |
| $SiF_4$ | -178.74 | -237.26 | -232.15 | -278.62 |
| $SiCl_4$ | -154.9 | -256.67 | -218.55 | -322.53 |
| $SiBr_4$ | -158.23 | -274.55 | -189.59 | -289.41 |
| $GeF_4$ | -192.23 | -225.09 | -225.01 | -276.56 |
| $GeCl_4$ | -192.04 | -225.88 | -223.51 | -276.89 |
| $GeBr_4$ | -190 | -262.56 | -165.03 | -269.69 |
| $SnCl_4$ | -185.84 | -223.75 | -223.85 | -275.15 |

| | | | | |
|---|---|---|---|---|
| $AsF_5$ | -169.34 | -262.01 | -209.93 | -290.26 |
| $SbF_5$ | -175.09 | -259.16 | -212.4 | -294.61 |
| $SbCl_5$ | -174.26 | -295.8 | -167.69 | -284.63 |
| $TiCl_4$ | -192.36 | -281.6 | -157.77 | -281.93 |
| $TiI_4$ | -157.61 | -298.22 | -160.08 | -289.07 |
| $ZrCl_4$ | -167.13 | -296.27 | -109.27 | -239.58 |
| $NbCl_5$ | -186.04 | -278.41 | -191.56 | -302.87 |
| $TaCl_5$ | -178.12 | -289.48 | -198.89 | -318.01 |
| $WF_6$ | -181.38 | -282.59 | -99.39 | -234.5 |
| $[C(CH_3)_3]^+$ | -162.89 | -273.04 | -230.01 | -324.88 |
| $[Ph_3C]^+$ | -155.74 | -294.75 | -180.46 | -303.34 |
| $[Ge(C_6F_5)_3]^+$ | -145.1 | -323.86 | -196.81 | -330.78 |
| $[Ph_3Sn]^+$ | -169.5 | -277.88 | -188.74 | -299.78 |
| $[Et_3Si]^+$ | -160.29 | -257.16 | -232.49 | -321.29 |
| $Sb(C_6F_5)_4^+$ | -178.39 | -285.43 | -135.15 | -261.27 |
| $[BMes]_2^+$ | -128.39 | -314.54 | -155.6 | -300.29 |
| $Al(OR^F)_3/SiO_2$ | -176.44 | -263 | -188.17 | -284.42 |
| HB(*o*-Me-Cb)/$SiO_2$ | -152.94 | -268.77 | -159.79 | -297.6 |
| $Zn^{2+}$/BEA (T2T7) | -178.26 | -257.6 | -189.98 | -287.23 |
| $Zn^{2+}$/BEA (T7T7) | -164.11 | -298.76 | -109.89 | -240.2 |
| $Cu^+$/BEA (T7) | -195.04 | -254.04 | -155.16 | -258.5 |
| $Ag^+$/BEA (T7) | -187.87 | -227.95 | -195.13 | -272.63 |
| $S_2O_7^{2-}/ZrO_2$ | -122.72 | -316.2 | -155.29 | -325.97 |
| $In^+$/BEA (T7) | -189.25 | -275.05 | -107.09 | -218.6 |

**Table S7.5** NCS Analysis $\sigma_{22}^{para}$.

| Lewis Acid | $\sigma_{22}^{para}$ (P–O) | $\sigma_{22}^{para}$ (P–C) | $\sigma_{22}^{para}$ (P–C) | $\sigma_{22}^{para}$ (P–C) |
|---|---|---|---|---|
| - | -246 | -32 | -177 | -176 |
| $B(C_6F_5)_3$ | -164.73 | -41.52 | -155.72 | -197.54 |
| $BPh_3$ | -169.53 | -45.36 | -144.21 | -193.29 |
| $Al(C_6F_5)_3$ | -155.31 | -28.52 | -201.16 | -196.26 |
| $AlPh_3$ | -170.24 | -28.07 | -239.73 | -193.95 |
| $BF_3$ | -175.79 | -88.71 | -88.74 | -207.55 |
| $BCl_3$ | -166.53 | -92.53 | -92.62 | -210.17 |
| $BBr_3$ | -166.33 | -40.56 | -157.31 | -201.02 |
| $BI_3$ | -164.53 | -26.72 | -228.43 | -195.14 |
| $AlF_3$ | -177.2 | -33.75 | -174.45 | -199.01 |
| $AlCl_3$ | -177.33 | -25.93 | -215.25 | -197 |
| $AlBr_3$ | -177.08 | -26.59 | -209.75 | -200 |
| $AlI_3$ | -172.41 | -60.94 | -139.01 | -199.2 |
| $GaF_3$ | -174.9 | -34.52 | -174.05 | -200.75 |
| $GaCl_3$ | -175.57 | -28.31 | -197.25 | -201.01 |
| $GaBr_3$ | -152.7 | -28 | -199.46 | -200.9 |
| $GaI_3$ | -185.06 | -76.71 | -113.58 | -189.95 |
| $InCl_3$ | -175.6 | -91.52 | -92.66 | -204.76 |
| $In(OTf)_3$ | -165.59 | -28.51 | -218.92 | -183.66 |
| $BiCl_3$ | -204.78 | -37.53 | -159.2 | -190.92 |
| $BiI_3$ | -199.57 | -29.24 | -208.4 | -183.46 |
| $Bi(OTf)_3$ | -162.33 | -75.28 | -116.07 | -204.33 |
| $Al(OR^F)_3$ | -176.77 | -26.41 | -217.06 | -196.41 |
| $SO_3$ | -159.11 | -48.6 | -147.34 | -195.1 |
| B(*o*-Cb)$_3$ | -122.51 | -70.62 | -117.27 | -178.94 |
| HB(*o*-Me-Cb) | -155.91 | -29.93 | -218.03 | -198.19 |
| $SiF_4$ | -160.91 | -89.62 | -90.58 | -205.4 |
| $SiCl_4$ | -163.66 | -77.01 | -111.29 | -195.3 |
| $SiBr_4$ | -157.3 | -53.02 | -135.02 | -194.67 |
| $GeF_4$ | -174.1 | -90.27 | -90.3 | -207.23 |
| $GeCl_4$ | -179.33 | -91.08 | -92.71 | -204.69 |
| $GeBr_4$ | -179.79 | -55.76 | -148.51 | -200.84 |
| $SnCl_4$ | -165.7 | -93.59 | -93.5 | -206.2 |

| | | | | |
|---|---|---|---|---|
| $AsF_5$ | -160.69 | -68.27 | -115.05 | -199.89 |
| $SbF_5$ | -165 | -71.08 | -111.66 | -197.77 |
| $SbCl_5$ | -168.09 | -42.15 | -165.52 | -196.89 |
| $TiCl_4$ | -181.45 | -40.85 | -155.04 | -192.67 |
| $TiI_4$ | -156.89 | -39.09 | -177.92 | -196.24 |
| $ZrCl_4$ | -167.08 | -29.08 | -221.06 | -199.92 |
| $NbCl_5$ | -175.73 | -55.08 | -133.43 | -199.43 |
| $TaCl_5$ | -173.27 | -54.57 | -138.31 | -204.24 |
| $WF_6$ | -177.51 | -31.99 | -208.46 | -179.44 |
| $[C(CH_3)_3]^+$ | -157.29 | -80.88 | -110.57 | -207.63 |
| $[Ph_3C]^+$ | -156.84 | -48.37 | -170.86 | -192 |
| $[Ge(C_6F_5)_3]^+$ | -144.82 | -50.97 | -177.29 | -199.75 |
| $[Ph_3Sn]^+$ | -158.06 | -59.43 | -148.04 | -198.16 |
| $[Et_3Si]^+$ | -152.7 | -85.07 | -108 | -213.67 |
| $Sb(C_6F_5)_4^+$ | -178.61 | -33.77 | -184.84 | -192.31 |
| $[BMes]_2^+$ | -135.26 | -42.68 | -181.78 | -196.85 |
| $Al(OR^F)_3/SiO_2$ | -175.09 | -52.37 | -132.19 | -197.77 |
| HB(*o*-Me-Cb)/$SiO_2$ | -168.5 | -33.05 | -155.6 | -174.61 |
| $Zn^{2+}$/BEA (T2T7) | -174.46 | -59.51 | -126.51 | -202.44 |
| $Zn^{2+}$/BEA (T7T7) | -164.4 | -28.88 | -223.6 | -199.03 |
| $Cu^+$/BEA (T7) | -196.79 | -48.91 | -138.61 | -187.78 |
| $Ag^+$/BEA (T7) | -193.24 | -81.84 | -100.15 | -184.04 |
| $S_2O_7^{2-}/ZrO_2$ | -122.01 | -23.03 | -209.61 | -186.42 |
| $In^+$/BEA (T7) | -188.94 | -28.01 | -205.9 | -194.51 |

**Table S7.6** NCS Analysis $\sigma_{33}^{para}$.

| Lewis Acid | $\sigma_{33}^{para}$ (P–O) | $\sigma_{33}^{para}$ (P–C) | $\sigma_{33}^{para}$ (P–C) | $\sigma_{33}^{para}$ (P–C) |
|---|---|---|---|---|
| - | 0 | -194 | -180 | -180 |
| $B(C_6F_5)_3$ | 0.06 | -193.7 | -181.28 | -197.54 |
| $BPh_3$ | 0.26 | -201.83 | -178.39 | -193.29 |
| $Al(C_6F_5)_3$ | 0.03 | -198.33 | -190.92 | -196.26 |
| $AlPh_3$ | 0 | -193.86 | -194.85 | -193.95 |
| $BF_3$ | -0.08 | -186.02 | -185.98 | -207.55 |
| $BCl_3$ | -0.21 | -188.81 | -188.69 | -210.17 |
| $BBr_3$ | -0.18 | -185.36 | -194.37 | -201.02 |
| $BI_3$ | -0.16 | -192.39 | -200.16 | -195.14 |
| $AlF_3$ | 0.01 | -184.12 | -195.24 | -199.01 |
| $AlCl_3$ | 0 | -196.86 | -191.34 | -197 |
| $AlBr_3$ | 0 | -189.46 | -197.56 | -200 |
| $AlI_3$ | 0 | -189.66 | -202.9 | -199.2 |
| $GaF_3$ | 0.07 | -180.65 | -194.12 | -200.75 |
| $GaCl_3$ | 0.05 | -185.06 | -194.58 | -201.01 |
| $GaBr_3$ | 0 | -185.55 | -194.98 | -200.9 |
| $GaI_3$ | -0.02 | -194.63 | -199.41 | -189.95 |
| $InCl_3$ | 0.07 | -186.89 | -186.69 | -204.76 |
| $In(OTf)_3$ | 0.1 | -195.48 | -195.66 | -183.66 |
| $BiCl_3$ | 0 | -196.19 | -185.47 | -190.92 |
| $BiI_3$ | 0 | -185.01 | -204.49 | -183.46 |
| $Bi(OTf)_3$ | 0 | -190.2 | -198.29 | -204.33 |
| $Al(OR^F)_3$ | 0 | -197.95 | -197.28 | -196.41 |
| $SO_3$ | 0.04 | -183.58 | -187.76 | -195.1 |
| B(*o*-Cb)$_3$ | 0 | -177.97 | -191.38 | -178.94 |
| HB(*o*-Me-Cb) | 0.23 | -185.08 | -190.06 | -198.19 |
| $SiF_4$ | -0.18 | -179.3 | -187.35 | -205.4 |
| $SiCl_4$ | 0.01 | -201.15 | -196.99 | -195.3 |
| $SiBr_4$ | 0.06 | -186.86 | -192.11 | -194.67 |
| $GeF_4$ | -0.01 | -184.79 | -184.84 | -207.23 |
| $GeCl_4$ | 0.06 | -186.47 | -185.74 | -204.69 |
| $GeBr_4$ | 0.04 | -179.71 | -193.84 | -200.84 |

| | | | | |
|---|---|---|---|---|
| $SnCl_4$ | 0.23 | -181.88 | -181.94 | -206.2 |
| $AsF_5$ | 0.1 | -186 | -192.91 | -199.89 |
| $SbF_5$ | -0.03 | -182.73 | -193.29 | -197.77 |
| $SbCl_5$ | -0.03 | -191.66 | -193.17 | -196.89 |
| $TiCl_4$ | -0.11 | -185.91 | -193.71 | -192.67 |
| $TiI_4$ | 0 | -194.16 | -196.72 | -196.24 |
| $ZrCl_4$ | 0.06 | -191.16 | -205.21 | -199.92 |
| $NbCl_5$ | -0.11 | -190.86 | -195.17 | -199.43 |
| $TaCl_5$ | -0.08 | -191.9 | -191.75 | -204.24 |
| $WF_6$ | 0.1 | -180.27 | -199.85 | -179.44 |
| $[C(CH_3)_3]^+$ | -0.01 | -194.27 | -195.04 | -207.63 |
| $[Ph_3C]^+$ | 0.43 | -197.1 | -194.67 | -192 |
| $[Ge(C_6F_5)_3]^+$ | 0 | -203.35 | -203.68 | -199.75 |
| $[Ph_3Sn]^+$ | 0.14 | -187.31 | -203.47 | -198.16 |
| $[Et_3Si]^+$ | 0 | -194.25 | -193.28 | -213.67 |
| $Sb(C_6F_5)_4^+$ | 0 | -191.38 | -191.79 | -192.31 |
| $[BMes]_2^+$ | 0.01 | -186.28 | -217.08 | -196.85 |
| $Al(OR^F)_3/SiO_2$ | 0 | -195.14 | -190.79 | -197.77 |
| HB(*o*-Me-Cb)/$SiO_2$ | 0.04 | -197.57 | -196.14 | -174.61 |
| $Zn^{2+}$/BEA (T2T7) | 0.01 | -191.08 | -186.09 | -202.44 |
| $Zn^{2+}$/BEA (T7T7) | 0.06 | -193.18 | -209.17 | -199.03 |
| $Cu^+$/BEA (T7) | -0.09 | -192.04 | -206.74 | -187.78 |
| $Ag^+$/BEA (T7) | -0.01 | -198.71 | -182.06 | -184.04 |
| $S_2O_7^{2-}/ZrO_2$ | 0 | -186.87 | -192.02 | -186.42 |
| $In^+$/BEA (T7) | -0.02 | -185.39 | -188.94 | -194.51 |

## References


(1) Beckett, M. A.; Brassington, D. S.; Coles, S. J.; Hursthouse, M. B. Lewis acidity of tris(pentafluorophenyl)borane: crystal and molecular structure of B(C6F5)3·OPEt3. *Inorg. Chem. Commun.* **2000**, *3*, 530-533.

(2) Halbert, S.; Copéret, C.; Raynaud, C.; Eisenstein, O. Elucidating the Link between NMR Chemical Shifts and Electronic Structure in d0 Olefin Metathesis Catalysts. *Journal of the American Chemical Society* **2016**, *138*, 2261-2272.

(3) Kolganov, A. A.; Gabrienko, A. A.; Stepanov, A. G. The DFT Approach to predict 13C NMR chemical shifts of hydrocarbon species adsorbed on Zn-modified zeolites. *Phys. Chem. Chem. Phys.* **2022**, *24*, 22241-22249.

(4) Jammee, R.; Kolganov, A.; Groves, M. C.; Pidko, E. A.; Sydora, O. L.; Conley, M. P. C− H Bond Activation by Sulfated Zirconium Oxide is Mediated by a Sulfur-Centered Lewis Superacid. *Angewandte Chemie International Edition* **2025**, e202421699.

(5) Schubert, U. Clusters with a Zr6O8 core. *Coordination Chemistry Reviews* **2022**, *469*, 214686.

(6) Pracht, P.; Bohle, F.; Grimme, S. Automated exploration of the low-energy chemical space with fast quantum chemical methods. *Physical Chemistry Chemical Physics* **2020**, *22*, 7169-7192.

(7) Bannwarth, C.; Ehlert, S.; Grimme, S. GFN2-xTB—An Accurate and Broadly Parametrized Self-Consistent Tight-Binding Quantum Chemical Method with Multipole Electrostatics and Density-Dependent Dispersion Contributions. *Journal of Chemical Theory and Computation* **2019**, *15*, 1652-1671.

(8) a) Mardirossian, N.; Head-Gordon, M. ωB97M-V: A combinatorially optimized, range-separated hybrid, meta-GGA density functional with VV10 nonlocal correlation. *The Journal of Chemical Physics* **2016**, *144*; b) Grimme, S.; Antony, J.; Ehrlich, S.; Krieg, H. A consistent and accurate ab initio parametrization of density functional dispersion correction (DFT-D) for the 94 elements H-Pu. *J. Chem. Phys.* **2010**, *132*;c)Grimme, S.; Ehrlich, S.; Goerigk, L. Effect of the damping function in dispersion corrected density functional theory. *Journal of Computational Chemistry* **2011**, *32*, 1456-1465; d)Weigend, F.; Ahlrichs, R. Balanced basis sets of split valence, triple zeta valence and quadruple zeta valence quality for H to Rn: Design and assessment of accuracy. *Physical Chemistry Chemical Physics* **2005**, *7*, 3297-3305.

(9) Neese, F. The ORCA Program System. *Wiley Interdisciplinary Reviews: Computational Molecular Science* **2012**, *2*, 73–78.

(10) Repisky, M.; Komorovsky, S.; Kadek, M.; Konecny, L.; Ekström, U.; Malkin, E.; Kaupp, M.; Ruud, K.; Malkina, O. L.; Malkin, V. G. ReSpect: Relativistic spectroscopy DFT program package. *The Journal of Chemical Physics* **2020**, *152*, 184101.

(11) Adamo, C.; Barone, V. Toward reliable density functional methods without adjustable parameters: The PBE0 model. *The Journal of Chemical Physics* **1999**, *110*, 6158-6170.

(12) Jensen, F. Segmented Contracted Basis Sets Optimized for Nuclear Magnetic Shielding. *Journal of Chemical Theory and Computation* **2015**, *11*, 132-138.

(13) Jensen, F. Polarization consistent basis sets: Principles. *The Journal of Chemical Physics* **2001**, *115*, 9113-9125.

(14) a) Dyall, K. G. Relativistic double-zeta, triple-zeta, and quadruple-zeta basis sets for the 4d elements Y–Cd. *Theoretical Chemistry Accounts* **2007**, *117*, 483-489; b) Dyall, K. G.

Relativistic double-zeta, triple-zeta, and quadruple-zeta basis sets for the 5d elements Hf–Hg. *Theoretical Chemistry Accounts* **2004**, *112*, 403-409.

(15) Glendening, E. D.; Landis, C. R.; Weinhold, F. NBO 7.0: New vistas in localized and delocalized chemical bonding theory. *Journal of Computational Chemistry* **2019**, *40*, 2234-2241.

(16) a) Franzke, Y. J.; Treß, R.; Pazdera, T. M.; Weigend, F. Error-consistent segmented contracted all-electron relativistic basis sets of double- and triple-zeta quality for NMR shielding constants. *Physical Chemistry Chemical Physics* **2019**, *21*, 16658-16664; b) Pollak,P.; Weigend, F. Segmented Contracted Error-Consistent Basis Sets of Double- and Triple-ζ Valence Quality for One- and Two-Component Relativistic All-Electron Calculations. *Journal of Chemical Theory and Computation* **2017**, *13*, 3696-3705.

(17) Bohmann, J. A.; Weinhold, F.; Farrar, T. C. Natural chemical shielding analysis of nuclear magnetic resonance shielding tensors from gauge-including atomic orbital calculations. *The Journal of Chemical Physics* **1997**, *107*, 1173-1184.

(18) van Lenthe, E.; Snijders, J. G.; Baerends, E. J. The zero-order regular approximation for relativistic effects: The effect of spin–orbit coupling in closed shell molecules. *The Journal of Chemical Physics* **1996**, *105*, 6505-6516.

(19) Rolfes, J. D.; Neese, F.; Pantazis, D. A. All-electron scalar relativistic basis sets for the elements Rb–Xe. *Journal of Computational Chemistry* **2020**, *41*, 1842-1849.

(20) a) Kühne, T. D.; Iannuzzi, M.; Del Ben, M.; Rybkin, V. V.; Seewald, P.; Stein, F.; Laino, T.; Khaliullin, R. Z.; Schütt, O.; Schiffmann, F.; Golze, D.; Wilhelm, J.; Chulkov, S.; Bani-Hashemian, M. H.; Weber, V.; Borštnik, U.; Taillefumier, M.; Jakobovits, A. S.; Lazzaro, A.; Pabst, H.; Müller, T.; Schade, R.; Guidon, M.; Andermatt, S.; Holmberg, N.; Schenter, G. K.; Hehn, A.; Bussy, A.; Belleflamme, F.; Tabacchi, G.; Glöß, A.; Lass, M.; Bethune, I.; Mundy, C. J.; Plessl, C.; Watkins, M.; VandeVondele, J.; Krack, M.; Hutter, J. CP2K: An electronic structure and molecular dynamics software package - Quickstep: Efficient and accurate electronic structure calculations. *The Journal of Chemical Physics* **2020**, *152*, 194103; b) Hutter, J.; Iannuzzi, M.; Schiffmann, F.; VandeVondele, J. cp2k: atomistic simulations of condensed matter systems. *WIREs Computational Molecular Science* **2014**, *4*, 15-25.

(21) Clark, S. J.; Segall, M. D.; Pickard, C. J.; Hasnip, P. J.; Probert, M. I. J.; Refson, K.; Payne, M. C. First principles methods using CASTEP. **2005**, *220*, 567-570.

(22) Tran, F.; Hutter, J. Nonlocal van der Waals functionals: The case of rare-gas dimers and solids. *The Journal of Chemical Physics* **2013**, *138*.

(23) Pickard, C. J.; Mauri, F. All-Electron Magnetic Response with Pseudopotentials: NMR Chemical Shifts. *Physical Review B* **2001**, *63*, 245101.

(24) Hans Reich's Collection. NMR Spectroscopy. https://organicchemistrydata.org/hansreich/resources/nmr/?index=nmr_index%2Finfo&page=nmr-content%2F.

(25) Sivaev, I. B.; Bregadze, V. I. Lewis acidity of boron compounds. *Coord. Chem. Rev.* **2014**, *270-271*, 75-88.

(26) Mummadi, S.; Kenefake, D.; Diaz, R.; Unruh, D. K.; Krempner, C. Interactions of Verkade's Superbase with Strong Lewis Acids: From Labile Mono- and Binuclear Lewis Acid–Base Complexes to Phosphenium Cations. *Inorg. Chem.* **2017**, *56*, 10748-10759.

(27) Erdmann, P.; Greb, L. What Distinguishes the Strength and the Effect of a Lewis Acid: Analysis of the Gutmann–Beckett Method. *Angew. Chem., Int. Ed.* **2022**, *61*, e202114550.

(28) Cheng, F.; Cheng, F. Gallium(III) halide complexes with phosphines, arsines and phosphine oxides - a comparative study. *Polyhedron.* **2007**, *26*, 4147-4155.

(29) Akram, M. O.; Tidwell, J. R.; Dutton, J. L.; Martin, C. D. Tris(ortho-carboranyl)borane: An Isolable, Halogen-Free, Lewis Superacid. *Angew. Chem., Int. Ed.* **2022**, *61*, e202212073.

(30) Akram, M. O.; Tidwell, J. R.; Dutton, J. L.; Martin, C. D. Bis(1-Methyl-ortho-Carboranyl)Borane. *Angew. Chem., Int. Ed.* **2023**, *62*, e202307040.

(31) Yoder, C. H.; Yoder, C. H.; Agee, T. M.; Schaeffer, C. D.; Carroll, M. J.; Fleisher, A. J.; DeToma, A. S.; Yoder, C. H.; Yoder, C. H.; Agee, T. M.; Schaeffer, C. D.; Carroll, M. J.; Fleisher, A. J.; DeToma, A. S. Use of 73 Ge NMR Spectroscopy for the Study of Electronic Interactions. *Inorganic chemistry.* **2008**, *47*, 10765-10770.

(32) Großekappenberg, H.; Reißmann, M.; Schmidtmann, M.; Müller, T. Quantitative Assessment of the Lewis Acidity of Silylium Ions. *Organometallics* **2015**, *34*, 4952-4958.

(33) Pan, B.; Gabbaï, F. P. [Sb(C6F5)4][B(C6F5)4]: An Air Stable, Lewis Acidic Stibonium Salt That Activates Strong Element-Fluorine Bonds. *J. Am. Chem. Soc.* **2014**, *136*, 9564-9567.

(34) Samudrala, K. K.; Huynh, W.; Dorn, R. W.; Rossini, A. J.; Conley, M. P. Formation of a Strong Heterogeneous Aluminum Lewis Acid on Silica. *Angew. Chem., Int. Ed.* **2022**, *61*, e202205745.

(35) Samudrala, K. K.; Akram, M. O.; Dutton, J. L.; Martin, C. D.; Conley, M. P. Formation of Strong Boron Lewis Acid Sites on Silica. *Inorg. Chem.* **2024**, *63*, 4939-4946.

(36) Jammee, R.; Kolganov, A.; Groves, M. C.; Pidko, E. A.; Sydora, O. L.; Conley, M. P. C−H Bond Activation by Sulfated Zirconium Oxide is Mediated by a Sulfur-Centered Lewis Superacid. *Angew. Chem., Int. Ed.* **2024**, e202421699.
